# Internally Catalyzed Hydrogen Atom Transfer (*I-CHAT*)—A New Class of Reactions in Combustion Chemistry

**DOI:** 10.3390/molecules30030524

**Published:** 2025-01-24

**Authors:** Rubik Asatryan, Jason Hudzik, Venus Amiri, Mark T. Swihart

**Affiliations:** Department of Chemical and Biological Engineering and Center for Hybrid Rocket Exascale Simulation Technology (CHREST), University at Buffalo, The State University of New York, Buffalo, NY 14260, USA; jasonhud@buffalo.edu (J.H.); venoosam@buffalo.edu (V.A.)

**Keywords:** intramolecular catalysis, hydrogen atom transfer, dihydrogen catalysis, autoignition, chain-branching, ketohydroperoxides, paraffin-based hybrid rocket fuels

## Abstract

The current paradigm of low-T combustion and autoignition of hydrocarbons is based on the sequential two-step oxygenation of fuel radicals. The key chain-branching occurs when the second oxygenation adduct (OOQOOH) is isomerized releasing an OH radical and a key *ketohydroperoxide* (KHP) intermediate. The subsequent homolytic dissociation of relatively weak O–O bonds in KHP generates two more radicals in the oxidation chain leading to ignition. Based on the recently introduced intramolecular “catalytic hydrogen atom transfer” mechanism (*J. Phys. Chem.* 2024, *128*, 2169), abbreviated here as *I-CHAT*, we have identified a novel unimolecular decomposition channel for KHPs to form their classical isomers*—enol hydroperoxides* (EHP). The uncertainty in the contribution of enols is typically due to the high computed barriers for conventional (“direct”) keto–enol tautomerization. Remarkably, the *I-CHAT* dramatically reduces such barriers. The novel mechanism can be regarded as an *intramolecular* version of the *intermolecular* relay transfer of H-atoms mediated by an external molecule following the general classification of such processes (*Catal. Rev.-Sci. Eng.* 2014, *56*, 403). Here, we present a detailed mechanistic and kinetic analysis of the *I-CHAT*-facilitated pathways applied to *n*-hexane, *n*-heptane, and *n*-pentane models as prototype molecules for gasoline, diesel, and hybrid rocket fuels. We particularly examined the formation kinetics and subsequent dissociation of the γ-enol-hydroperoxide isomer of the most abundant pentane-derived isomer γ-C5-KHP observed experimentally. To gain molecular-level insight into the *I-CHAT* catalysis, we have also explored the role of the internal catalyst moieties using truncated models. All applied models demonstrated a significant reduction in the isomerization barriers, primarily due to the decreased ring strain in transition states. In addition, the longer-range and sequential H-migration processes were also identified and illustrated via a *combined double* keto–enol conversion of heptane-2,6-diketo-4-hydroperoxide as a potential chain-branching model. To assess the possible impact of the *I-CHAT* channels on global fuel combustion characteristics, we performed a detailed kinetic analysis of the isomerization and decomposition of γ-C5-KHP comparing *I-CHAT* with key alternative reactions—direct dissociation and Korcek channels. Calculated rate parameters were implemented into a modified version of the *n*-pentane kinetic model developed earlier using RMG automated model generation tools (*ACS Omega,* 2023, *8,* 4908). Simulations of ignition delay times revealed the significant effect of the new pathways, suggesting an important role of the *I-CHAT* pathways in the low-T combustion of large alkanes.

## 1. Introduction

Low-temperature combustion of hydrocarbons (<900 K) is a complex process primarily controlled by fuel-specific oxidation reactions, as opposed to the high-temperature processes, which are mostly governed by the breakdown of fuel molecules into small radical fragments prior to oxidation [1,2,3,4,5,6,7]. The key reactions of low-temperature autoignition of hydrocarbon–air mixtures involve the formation and decomposition of ketohydroperoxide (KHP) intermediates [3,5,8,9,10,11,12,13,14,15,16]. KHPs also play a central role in tropospheric oxidation and aerosol (secondary organic aerosol, SOA) formation processes [17,18]. Whereas much is known about the mechanism of low-T combustion processes in small models, more specific reaction channels are currently emerging for the practically important larger systems, providing new insights into the overall reaction mechanisms. One of these pathways actively studied in recent years includes the formation of highly oxygenated molecules (HOMs) via third- and higher-degree oxygenation reactions [17,18,19,20]). These and other prospective mechanisms can be particularly important in understanding the combustion mechanism of large and extra-large alkanes, such as paraffin wax, which is an important hybrid rocket fuel (HRF) [21,22,23,24,25,26]. Commercial paraffin waxes typically comprise a mixture of extra-large linear alkanes, with small amounts of branched alkanes (paraffin oil). An understanding of their detailed oxidation chemistry is important for improving hybrid rocket performance [24,26].

**Scheme 1:**
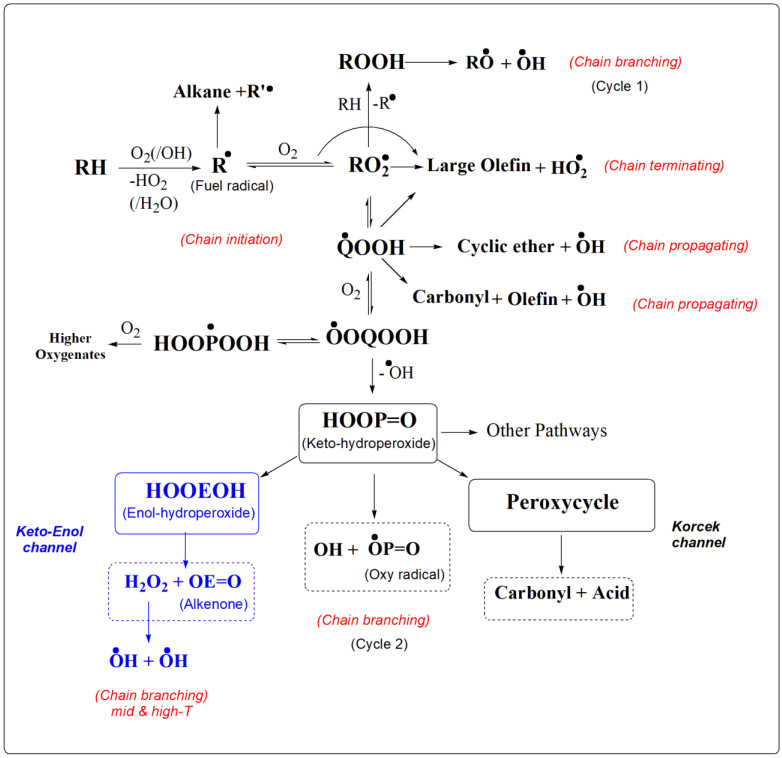
Oxidation model for large alkanes, including two novel KHP decomposition pathways—sequences of Korcek and keto–enol tautomerization (highlighted in blue) reactions. **Q**, **P**, and **E** core structures have, correspondingly, 1, 2, and 3 H atoms less than **R**; for pentane derivatives, they represent, correspondingly, C_5_H_11_-, C_5_H_10_-, C_5_H_9_-, and C_5_H_8_- groups.

**Scheme 2:**
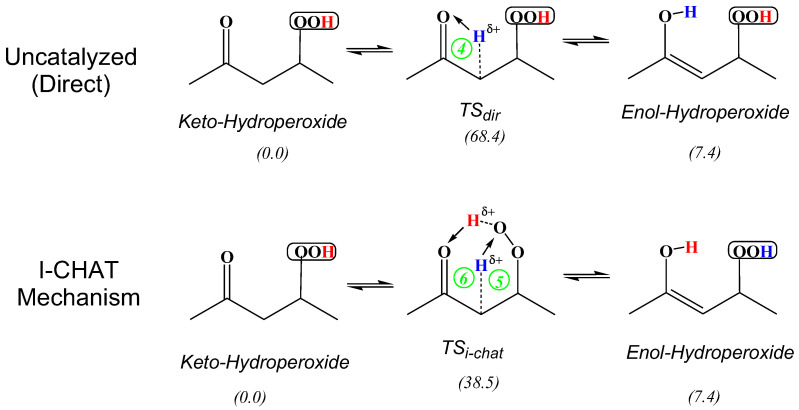
Internally catalyzed (intramolecular) hydrogen atom transfer (*I-CHAT*) mechanism versus *Direct* keto–enol tautomerization of the model pentane-γ-ketohydroperoxide (γ-KHP)—a key chain-branching agent of the combustion of traditional fuels. The internal catalyst group (here OOH) is highlighted. Relative enthalpies are provided in parenthesis. *I-CHAT* reduces the barrier by as much as 30 kcal/mol.

Scheme 1 provides the current view of the low-temperature combustion of large alkanes, involving sequential double oxygenation reactions of fuel radicals accounting for autoignition via the formation of key ketohydroperoxide (KHP) intermediates, O=POOH. KHPs further undergo homolytic dissociation of relatively weak O–O bonds to generate two active radicals in the oxidation chain to complete the main chain-branching event (*vide infra*). Scheme 1 also includes two recently discovered alternative unimolecular decomposition channels for KHPs—the Korcek mechanism [27] and the *I-CHAT* mechanism, which is highlighted in blue [8,15]. Both channels include an intricate isomerization of KHPs. In the first case, it leads to the formation and dissociation of a peroxycyclic intermediate, whereas the second case occurs via internally catalyzed keto–enol conversion (*I-CHAT* pathway in Scheme 2) and decomposition of enol hydroperoxide counterparts.

### 1.1. I-CHAT Mechanism

The keto–enol conversion depicted in Scheme 2 is a particular case of the general mechanism we have recently introduced [8,15]. It represents a new type of intramolecular isomerization reaction, which occurs via the synchronized (*internally catalyzed*) migration of hydrogen atoms, initially designated simply as “catalytic hydrogen atom transfer”—*CHAT*. Because the short *CHAT* acronym we employed previously is too general and often mistaken with metallocomplex-catalyzed processes, here, we place emphasis on its definitive intramolecular character by using the term “***internally catalyzed hydrogen atom transfer***”, abbreviated as ***I-CHAT***, which seems to represent the mechanism uniquely and adequately. We did not use the term “self-catalysis” either, because it is typically used in *bimolecular* processes as a synonym of autocatalysis, through which a product catalyzes reactions to convert substrates into products. In our case, however, one *intramolecular* H-migration act catalyzes another H-migration act, making it a double H-transfer within the same molecule. This is due to the “compensatory” H-migration phenomenon described in detail in [8].

Scheme 2 illustrates the *I-CHAT* mechanism applied to pentane 2,4-ketohydroperoxide (2,4-KHP, γ-C5-KHP, or γ-KHP). The catalyst moiety (here, an encircled peroxy group) transfers its H atom to the carbonyl group, simultaneously accepting a hydrogen atom of the skeletal α-methylene group, thereby mediating a tautomerization process, forming 2,4-enolhydroperoxide (γ-EHP) and regenerating the catalyst moiety. In contrast to the classical tautomerization via direct H-transfer, which occurs via a strained 4-member-ring TS and faces a high activation barrier of 61 kcal/mol (top part of the Scheme 2), the *I-CHAT* mechanism occurs through a TS with two enlarged (5 and 6-membered) and fused ring structures, thus markedly reducing the conversion barrier due to the split. Therefore, the new mechanism can be characterized as a *relay* transfer of H-atoms, consistent with the general systematization of the molecular catalysis processes provided earlier [28,29], albeit it occurs in an intramolecular manner.

Generally, the *I-CHAT* mechanism belongs to the *intramolecular catalysis* class of reactions following the IUPAC guidelines: “Intramolecular Catalysis is the acceleration of a chemical transformation at one site of a molecular entity through the involvement of another functional (’catalytic’) group in the same molecular entity, without that group appearing to have undergone change in the reaction product” [30]. The novel mechanism, indeed, occurs via the intramolecular transfer of hydrogen atoms mediated by a functional group (*I-CHAT* group, moiety), such as -CH_2_OH-, HOO-, -COOH, -SH, and -NH_2_. However, it strongly occurs in a molecular entity, without the involvement of a second-party agent/co-reagent, which normally occurs in traditional intramolecular processes involving bimolecular reactions mediated by, e.g., water molecules in intramolecular hydrolysis [31] or acids in some isomerization processes [32], with the catalyst group being in the molecule-substrate. On the contrary, *I-CHAT* is a strictly unimolecular *intramolecular* process, proceeding without the involvement of any external agent or co-reagent; instead, the embedded catalyst group catalyzes the reaction, as illustrated in the bottom portion of Scheme 2 representing reactions of γ-KHPs, where the hydroperoxy and carbonyl functionalities are separated by a methylene linkage.

The *I-CHAT* catalyst group promotes various interconversion processes, such as keto–enol and imino–amino tautomerization, double-bond shift, and cyclization, while recovering itself, as shown in Scheme 2. Thus, *I-CHAT* is a general process that proceeds via much lower energy barriers than alternative uncatalyzed (direct) isomerization reactions due primarily to the eased ring strains in transition states and can be relevant for a variety of systems and conditions. Some characteristic examples of *I-CHAT* facilitated processes with varied electronic and steric characteristics (polarity and non-rigidity) are provided in Section 2.1 (simple model results are combined in Table 1).

Internally catalyzed bond-exchange process can be regarded as an *intramolecular* version of the intermolecular relay transfer of H-atoms mediated using an external single molecule (molecular catalyst), e.g., dihydrogen, water, NH_3_, various carboxylic (primarily formic and acetic), and inorganic acids, as well as radicals [8].

Asatryan and Ruckenstein classified these intermolecular catalysis processes into five major reaction categories, illustrated in the simplest possible case of the H_2_-mediated reactions, called *dihydrogen catalysis* (DHC) [28,29]. The five suggested categories include: (**A**) dihydrogen-assisted relay transfer of H-atoms, (**B**) dihydrogen-assisted stepwise-relay transport of H-atoms/free valence, (**C**) dihydrogen-assisted proton transport, (**D**) dihydrogen-assisted dehydrogenation/hydrogenation, and (**E**) pre-activated dehydrogenation [28,29]. Following this systematization, the *I-CHAT* catalysis belongs within category **A**—relay transfer of hydrogen atoms (H-atom switch), since the *I-CHAT*-catalyst (moiety) simultaneously acts as a *relay* H-atom-donor and acceptor, although in an *intramolecular* manner. Note that two additional subcategories of the relay transfer mechanism of category **A** have also recently been identified [8].

Note that a broader systematization of molecular catalysis processes was provided by Francisco et al., considering only double H-bond transfer (viz. H-atom relay) processes in atmospheric chemistry [33]. It is also important to note that there are many other mechanisms, closely related to category **A**, suggested in the literature, such as a proton-relay mechanism widely employed in biochemistry, concerted biprotonic transfer, and proton pumps for the transport of protons across membranes, long known and intensively studied and speculated [34,35,36,37,38]. The key pieces of evidence for the popular water- and carboxylic (acetic) acid-catalyzed proton-relay keto–enol transformations, for instance, have been identified in early works by Bernasconi [37] and Song [38], respectively. However, in contrast to all these bimolecular and multi-molecular catalytic processes, *I-CHAT* is a genuine *unimolecular* and purely *intramolecular* process with no external source of H-atoms/protons being involved.

Thus, we have identified a novel low-energy unimolecular decomposition pathway for the key combustion intermediate KHPs leading to the formation of *enol hydroperoxides* (EHP)—the classical isomers of KHPs. We have tested their relevance in global combustion processes (Section 2.5). Even though the enols have recently gained much attention in the combustion community, the contribution of EHPs remains unknown and challenging due to the coupling of PESs for two unimolecular alternative reactions, as well as the high computed barriers for direct isomerization of corresponding KHPs. On the other hand, the novel, *I-CHAT*-based keto–enol tautomerization mechanism fundamentally increases the conversion rates of the KHPs.

In addition, the *I-CHAT* process can “spread over” within a large molecule given that other reactive centers, such as a carbonyl group, are available in the vicinity because the catalyst moiety is regenerated/reinstated each time, as detailed in Section 2.2. Apparently, such a consecutive act can primarily occur in pre-activated systems (chemically or photochemically) as in the case of ketohydroperoxide intermediates formed from double chemical activation of fuel radicals by two O_2_ molecules (Scheme 1). Perhaps, some other “double activation” and non-Boltzmann processes can also promote such reactions [39,40,41]. Furthermore, longer-range *I-CHAT* processes can also occur during the combustion of large hydrocarbons, depending on the dimension and electronic properties of the *I-CHAT*—catalyst groups and the H-donor/acceptor centers.

### 1.2. Low-T Chain Branching

The currently accepted mechanism of low-T combustion and the autoignition of hydrocarbons (large alkanes) illustrated in Scheme 1 is based on the sequential two-step oxygenation of fuel radicals accounting for autoignition. Scheme 1 also includes the Korcek mechanism, as well as the new *I-CHAT* reaction mechanism, highlighted in blue, as alternative unimolecular decomposition reactions of KHPs [8,15,27]. The addition of the first O_2_ molecule to the fuel radical (R), typically produced via a radical attack on the fuel molecule, forms a peroxy radical RO_2_. The peroxy radical per se can abstract a hydrogen atom from an H-donor (typically a fuel molecule) to form a hydroperoxide, ROOH, or undergo an intramolecular H-migration—abstraction process (tail-biting isomerization)—to generate a carbon-centered hydroperoxyalkyl radical commonly denoted as QOOH. The direct dissociation of QOOH is a chain-propagating process producing an OH radical and QO molecular fragment (cyclic ether or olefin + carbonyl).

Early investigations assumed that the dissociation of the peroxy bond in ROOH (Cycle 1 in Scheme 1) to form two active radicals RO and OH is the key low T chain-branching process [1,2,3,4,5,6,7,42,43,44,45,46]. However, the initial step in this sequence (H-abstraction from a fuel molecule by an alkylperoxy radical), is typically too slow to explain experimental observations, as argued by Taatjes and co-workers (2009), providing fundamental evidence for the long-hypothesized (see, e.g., [1,6,8,22]) alternative second oxygenation pathways, based on experiments on the cyclohexyl + O_2_ reaction system [47]. Because the PES for O_2_QOOH had not yet been explored at the time, the offered evidence was limited to the interpretation of time-resolved experiments suggesting the existence of very low-lying exit channels; however, it was supported by the emerging first-principles-based predictions on such low-lying branching channels in double oxygenation of pentyl radicals [48,49] (see also [11]), as highlighted by the authors [47] and noted by others [7].

Thus, the critical chain branching events occur through the addition of the second O_2_ to the QOOH radical forming an oxygen-centered hydroperoxyalkyl peroxy OOQOOH radical adduct (Scheme 1). The subsequent decomposition of OOQOOH via intramolecular H-abstraction through the peroxy radical center (second tail biting event), preferably at the proximal carbon atom bearing the hydroperoxy group, generates the first OH-radical in the oxidation chain and forms a key chain-branching agent—ketohydroperoxide, O=POOH (P has one less H-atom than Q). The nearly instant dissociation of the metastable dihydroperoxy alkyl radical-intermediate is due to the formation of the essentially unbound OH- group [11]. On the other hand, the energetically less preferred H-abstraction from an alternative C-center produces relatively more stable dihydroperoxy alkyl radicals [3,10,11] (HOOPOOH in Scheme 1), which can further add O_2_ and lead to the formation of highly oxygenated molecules (HOM) actively studied in recent years, particularly in the context of atmospheric aerosol formation [17,19]. Further cleavage of the relatively weak O–O bond in ketohydroperoxides (BDE of 40–45 kcal/mol [13,14,43]) releases a second OH radical and forms another open-shell reactive species—keto-alkoxy radical, O=PO^●^, thus concluding the low-T chain branching process. Overall, three radicals produced in the oxidation chain per one OH-radical consumed to produce an initial fuel radical sharply increases the fuel reactivity and triggers ignition/explosion. The fundamental aspects of these processes based on the analysis of the combined R + O_2_ and QOOH + O_2_ potential energy surfaces (PES) have been initially reported for model pentyl and propyl radicals [10,11,12,48,49], which showed that O_2_QOOH isomerization to KHP + OH is the dominant chain branching reaction at low temperatures (below 1000 K). Subsequent theoretical studies brought in an understanding of the various aspects of the intramolecular isomerization of OOQOOH [50,51,52,53,54,55,56,57,58], formation of highly oxygenated molecules (HOM) [17,18,19,20], as well as bimolecular reactions involving KHPs [56].

Ketohydroperoxides (KHPs) were identified and characterized for the first time in the early 1990s by Sahetchian and coworkers [9,54,55,57,59,60,61] in hexane, heptane, and dodecane oxidation using gas chromatography and direct-injection mass spectrometry in a reactor and CFR engine. These studies showed that KHPs are formed by isomerization reactions and play a key role in combustion chemistry and tropospheric oxidation. However, they gained a fast-growing interest in around 2010 prompted by emerging new detection techniques primarily by Battin-Leclerc and Taatjes and coworkers performing breakthrough studies [3,5,62,63,64,65,66,67,68]. These experiments on the formation and decomposition of diverse KHPs also stemmed from the above-noted first principles-based fundamental theoretical predictions [10,11,12,48,49,50].

Subsequently, ketohydroperoxides have been observed in a variety of low-T combustion processes involving hydrocarbons and oxygenated fuels [69,70,71,72,73]. Notably, the KHPs have recently been applied as markers of low-temperature kinetics by Dryer, Avedisian, and coworkers to demonstrate the multistage *cool flame* behavior of primary reference fuel (PRF) droplets [74]. This study seems to be the most relevant analysis of the combustion of paraffin wax as a hybrid rocket fuel (HRF), where the combustion of HRFs occurs (at variable conditions) via liquid layer formation, fuel entrainment, droplet formation, and evaporation [22,23,24,25,26].

In conventional alkane oxidation models, the simple O–O bond fission had been the only dissociation pathway for KHP numerical simulations [2,3,6,10,11]. However, recent studies have shown that other channels can compete with this chain-branching event, such as the bimolecular reactions of KHP [34,59,67,68], as well as the unimolecular Korcek reaction [27], in which the decomposition of KHP forms carboxylic acid and carbonyl products. The Korcek pathway was the most significant recent contribution to the mechanism of low-T combustion (Scheme 1) theoretically described by Jalan et al. [27]. It is an alternative to the simple unimolecular dissociation of KHP and occurs via its isomerization to a peroxy-cyclic intermediate. The relevance of the Korcek pathway has recently been evidenced (corroborated) by time-resolved experiments by Taatjes and co-workers, confirming the formation of correlated “Korcek pairs” using isotopically labeled *n*-butane [75]. The authors, however, concluded that even though the Korcek mechanism explains the formation of a significant part of the experimentally observed carboxylic acids and carbonyl products, it is not a chain-branching process that can affect autoignition. This contrasts with our *I-CHAT* mechanism for the decomposition of KHPs (highlighted in blue in Scheme 1), which suggests the formation of more diverse products, including the key mid-temperature, chain-branching agent H_2_O_2_.

Overall, the *I-CHAT* is a new intramolecular isomerization mechanism that provides low-energy pathways for the formation and decomposition of enol hydroperoxide (EHP) isomers of KHPs.

Here, we explore in detail the role of the novel internally catalyzed hydrogen atom transfer (*I-CHAT*) mechanism in combustion processes. The unimolecular decomposition of large model KHPs, such as *n*-heptane and *n*-hexane along with *n*-pentane are of special importance for understanding the combustion of non-rigid large hydrocarbons, such as paraffin-wax hybrid-rocket fuels with a multitude of stereochemical transformation channels. We also provide more extended kinetic analysis of *I-CHAT* reactions to study their relevance in the combustion of *n*-alkanes represented by a *n*-pentane ketohydroperoxide model—γ-C5-KHP, which is the most abundant isomer of the KHPs, derived from pentane, in which C=O and HOO groups are separated by a methylene linkage.

To assess the importance of *I-CHAT* reactions in combustion processes, we have incorporated rate parameters calculated in Section 2.4 into a chemical kinetic model for the combustion of *n*-pentane generated previously using RMG automated mechanism generation software [21]. The simulation results on the ignition delay times (IDTs) compared with literature data are discussed in Section 2.5, showing the significant role of the *I-CHAT* pathways in the low-T combustion of large alkanes.

In the next section, we briefly describe methods used for the first principles’ modeling and analysis of the potential energy surfaces (PESs) of the internally catalyzed (intramolecular) hydrogen atom transfer pathways, as well as the calculation of rate parameters, along with the generation of modified kinetic models and simulation results. A detailed analysis of the several characteristic small model reactions facilitated by this mechanism is further provided.

## 2. Results and Discussion

Keto–enol tautomerization plays several essential roles in combustion chemistry due to the different reactivity of tautomers. Enols are significant combustion intermediates important for flame chemistry [76,77,78,79,80]. The keto tautomer (isomers of carbonyl compounds—ketones/aldehydes) is usually strongly favored, especially in polar liquids [81,82,83,84,85,86,87].

*I-CHAT* is a new mechanism for the energetically more facile formation of typically less stable enols to explain their roles and unexpected occurrence in combustion (in flame [88,89,90,91,92] and atmospheric processes (missing enols [93]).

### 2.1. Keto–Enol Tautomerization and Skeletal Double Bond Shift Isomerization

The uncatalyzed (direct) H-transfer (keto–enol tautomerization) in the gas phase is typically a high-energy process encountering a sizable activation barrier due to the formation of the small (here, four-membered) strained-ring transition states (top part of the Scheme 2). However, various acid-base catalysts, biocatalysts (enzymes), and molecular catalysts are known to assist tautomerization and reduce the barrier. Solvents can also affect these processes in the condensed phase depending on the polarity of the TS. Polar media typically favors the keto form (see, e.g., [80,94]) due to the higher polarity of keto forms in polar liquids. The di-keto form of acetylacetone, for instance, predominates both in water and DMSO, while the enol form is more stable in non-polar CCl_4_ in accordance with corresponding dipole moments [88]. At very low temperatures, in liquids and the solid state, tunneling also significantly impacts the rate parameters [85,86].

**Scheme 3:**
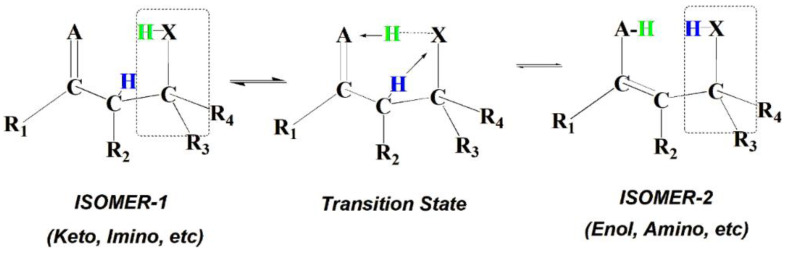
Generalized sketch of the *I-CHAT* bond-exchange mechanism relevant to conventional (e.g., keto–enol and imino–amino tautomerization, where **A** is O or NH, respectively; cf. Scheme 2), and skeletal double-bond shift isomerization (**A** ≡ CH_2_). **A** symbolizes the adjacent H-acceptor group of the molecule undergoing conversion. **XH** represents an electronegative heteroatom containing the H-donor/acceptor ambivalent group (basically, the core of the *I-CHAT* catalyst), such as -OOH, -SH, -COOH, -OH, or -NH_2_. The broader catalyst moiety responsible for accessibility to **A** is encircled.

A general schematic of the *I-CHAT* processes involving two specific, topologically close processes—the tautomerization and carbon-carbon double bond shift (DBS) isomerization—is provided in Scheme 3. For simplicity, only forward vibration modes in TS are shown for these reversible reactions. A double-bond shift process facilitated using *I-CHAT* catalysis can occur when **A** is a methylene group.

Apparently, DBS can even be more relevant at elevated temperatures and in flames, where PAHs and soot particles are formed because of its relatively higher homolytic dissociation barrier and thus stability. Tautomerization is known to occur with polar molecules and ions containing functional groups that are at least weakly acidic. Even though the carbon-chain isomerization via a double-bond shift (Scheme 3) is formally identical to that for the *I-CHAT* keto–enol tautomerization (when **A** is CH_2_ group versus O-atom), it encounters a significantly higher barrier of activation, mainly due to the lack of electrostatic stabilizing interactions in the TS. On the other hand, the π-electrons involved in the TS also allow the intramolecular DBS reaction, as in the case of *intermolecular* (bimolecular) catalysis processes [28,29].

***Effect of the Catalyst Moiety.*** The *I-CHAT* mechanism can potentially involve a variety of “*I-CHAT*-catalyst” groups, such as OOH, -CH_2_OH, -COOH, and -SH. Therefore, we also explored their catalytic efficiency depending on the electronic and structural characteristics of those groups. Some groups are relatively less thermostable than others and prone to dissociate at lower temperatures, such as a peroxy group containing a relatively weak O–O bond. Therefore, the *I-CHAT* mechanism can potentially operate at different temperature ranges depending on the nature of the catalyst moiety. However, the catalyst group might also somewhat lack the steric flexibility and “stretchability” of the peroxy group as an *I-CHAT*-catalyst. Replacing the hydroperoxy moiety with a hydroxymethyl (-CH_2_OH) group with an added methylene linkage to maintain the steric accessibility and flexibility of the OH- group, for instance, does not introduce any significant changes in reaction barriers compared to that of the HOO catalyst (*vide infra*), yet it is more stable, and direct dissociation at lower temperatures is not possible. Apparently, this does not necessarily mean that the OH-dissociation threshold in KHPs containing OOH- groups is too low. In fact, it is still substantially higher (by 6–8 kcal/mol) than the barrier height for the *I-CHAT* conversion, which makes the latter channel competitive (see details in Section 2.4).

Based on the above discussion, a series of simple *I-CHAT* reaction models were constructed for the bond-exchange processes to model larger systems involving different **XH** groups maintaining the C3 backbones. The results are presented in Table 1.

The smallest relevant model of the KHP that undergoes *I-CHAT* conversion is 1-hydroperoxy-2-ethanone (HOOCH_2_CHO)—the hydroperoxy derivative of acetaldehyde. However, it does not contain a CH_2_ linkage and, thus, it is not included in Table 1. The catalytic conversion barrier, as expected from the increased strain of the small TS ring, is much higher than those for the analogous extended models provided in Table 1 at ca. 57.7 kcal/mol (vs. 75.5 kcal/mol for the direct H-transfer).

**Table 1:** Internally catalyzed tautomerization of skeletal (truncated) tri-carbon models.

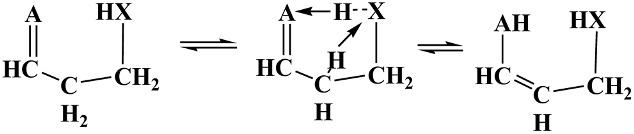										
**=A**	**χA ^(a)^**	**XH**	***q* (A) ^(b)^**	***q* (X) ^(b)^**	**ΔG^#^_I-CHAT_**	***ν*_1_ ^(c)^,** **cm^−1^**	**ΔG^#^_dir_**	**ΔG_r_**	***q* (H) ^(b)^**	***q* (C) ^(b)^**
S	2.58	OOH	−0.287	−0.332	34.25	−1162.2	55.97	−1.95	0.261	−0.333
NH	3.04	OOH	−0.673	−0.488	37.57	−1083.8	65.97	−0.14	0.358	−0.043
NH	3.04	SH	−0.757	−0.302	38.30	−1151.5	65.87	−2.14	0.173	−0.137
O	3.44	OOH	−0.728	−0.457	39.61	−1481.4	68.10	6.07	0.356	−0.06
O	3.44	CH_2_OH	−0.755	−0.657	40.17	−1397.0	69.17	5.56	0.276	−0.345
O	3.44	COOH	−0.701	−0.544	45.41	−1456.1	70.73	7.83	0.375	−0.354
O	3.44	SH	−0.702	−0.298	49.06	−1295.9	70.75	6.17	0.200	−0.239
CH_2_	2.55	OOH	−0.714	−0.451	59.35	−1681.2	75.75	−1.08	0.330	−0.025

^(a)^ Pauling electronegativity of H-acceptor atom **A**, ^(b)^ Mulliken partial charges on **A**, **X**, and migrating **H** atoms; ^(c)^ imaginary frequency for TS_I-CHAT_. Gibbs energies are in kcal/mol, partial charges in electrons.

Table 1 provides some energetic and electronic characteristics of models, such as activation Gibbs energy barriers for *I-CHAT*-facilitated (ΔG^#^_I-CHAT_) and direct (ΔG^#^_dir_) reactions, as well as reaction energies (ΔG_r_), along with the Pauling electronegativity of the acceptor centers (χA) and the partial Mulliken charges on reactive A, X, and carbon centers (*q*) of the keto forms (here, formal reagents). In Table 1, *q*(*C*) is the net charge on the carbon atom connected to the reaction center, which undergoes enolization.

A few general conclusions can be observed after an inspection of Table 1.

(1). Formally, an *I-CHAT* group consists of two reactive (double-centered) bonds such as O-O-H or CH_2_-O-O, involved in the TS to provide steric flexibility and orbital overlaps, except when a single bond is significantly longer to provide access to the acceptor site, as occurs in the case of the S–H bond. For instance, the hydroxymethyl (CH_2_OH) group as an I-CHAT-agent is sterically and energetically almost as effective as OOH (energy profiles are similar, and the barrier heights are very close: 39.61 vs. 40.17 kcal/mol, respectively), suggesting that the ring-strain indeed is the dominant factor in *I-CHAT* processes.
(2). The decrease in ring strain in the *I-CHAT* TS, relative to the TS for the direct isomerization, is primarily due to the splitting of a small TS-ring of the uncatalyzed (direct) reaction into two larger rings of the catalyzed reaction, as shown in Scheme 2. In addition, one intramolecular H-bond between a pair of donor and acceptor centers (XH…A) is converted into two H-bonded donor-acceptor motifs (AH…X and XH…A, Scheme 3). Therefore, the barrier height dependence on the electronic characteristics is not straightforward; rather, it varies with the nature of the different constituent rings. The electronic structure (judging from partial atomic charges or relative electronegativities) of the two H-acceptor and donating centers (χ_A_ and χ_X_) is, a priori, expected to play an important role, and their competition can be a key factor. Therefore, analyzing these factors can be useful in understanding the specific interactions during relay H-atom transfer.

As seen in Table 1, the Pauling electronegativity of the **A** acceptor centers (χ_A_) correlates with the partial charges on donor atoms. Surprisingly, the partial negative charges (electronegativity) of the **A** centers are inversely related with the barrier heights (comparisons are made among systems involving the same—here, OOH, *I-CHAT* groups—for consistency). Comparisons among other *I-CHAT* groups presented in Table 1 containing the same **A**-center also confirm this conjecture: when χX decreased, the barrier increased. This suggests that the simple electrostatic theory one could expect to be dominant in single H-bonding pairs is not sufficient to make definitive conclusions.

(3). Notably, the migrating H atom is more positively charged in enols than in the keto ground state, revealing the polar character of the H–OO bond as opposed to the C-H bond, which is more difficult to split. Thus, the more influential ring is the one involving the fission of the stronger C–H bond depending on the ability of the X-center to abstract the corresponding H-atom.
(4). When a sterically more flexible and polar group (OOH) is combined with a longer double bond of the acceptor site such as C=S, the barrier is reduced. This is in accordance with conclusions from Francisco and coworkers on intermolecular H-migration processes, where longer S=O bond forms stronger H-bonds [89].
(5). The barrier heights correlate with the topological properties of PESs. Particularly, an increase in the imaginary frequency in the TS correlates with the barrier heights among systems possessing the same XH catalyst group, e.g., OOH and SH in Table 1.
(6). The Gibbs energy of the reactions (ΔG_r_), as a merit of relative stability of reagents, also broadly correlates with the barrier heights when compared across similar groups, in accordance with the Bell–Evans–Polanyi kinetic rule [90,91,92].

### 2.2. Long-Range and Sequential I-CHAT Catalysis

As noted above, the keto–enol tautomerization may also occur between distant centers via the extended saddle-point structures provided by the *I-CHAT*-catalysis. The extent to which this occurs depends on the steric and electronic properties of the catalytic center and the accessibility of the other involved reactive centers. Generally, the process can also span the inter-chain regions (areas) as well. Moreover, the *I-CHAT* process can potentially occur more than once within the same molecule. Below are some examples for illustration.

***Sequential (Double) I-CHAT Models.*** The *I-CHAT* pathway can occur multiple times in a sequential manner provided that another similar reactive center is available in the vicinity to undergo isomerization, because the *I-CHAT*-moiety is recovered after each elementary act. Multifunctional hydroperoxides with zero, one, two, or more carbonyl groups have been shown to form key species during the oxidation of large alkanes (e.g., *n*-dodecane) in reactors and engines [61]. They were observed in the troposphere [68], cool flames [18], and combustion media [18,19,20]. There is also a variety of multi-carbonyl systems—natural products, such as alkyl malonates and poly(β-oxo)carboxylic acids (polyketides) important for pharmacology, for which keto–enol tautomerization is a key issue (see, e.g., [80]).

**Scheme 4:**
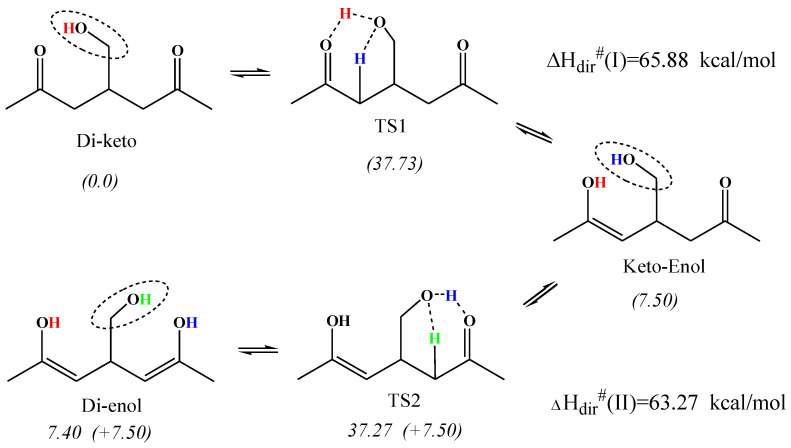
Double-*I-CHAT* tautomerization of the 2,6-diketo-4-hydroxymethyl heptane (4-hydroxymethyl-2,6-heptane-dione)—a KHP-model of the *n*-heptane combustion. The *I-CHAT*-catalyst (OH of the encircled CH_2_OH group) relay-transfers its H atom to the first carbonyl group to catalyze its tautomerization and recovers itself by receiving an H-atom from the α-carbon backbone; then, the same process occurs with the second carbonyl oxygen, again recovering the hydroxymethyl catalyst group. Relative to reagents, enthalpies are in kcal/mol.

Scheme 4 provides an example of the sequential double *I-CHAT* reactions applied to a ketohydroperoxide model heptane-2,6-diketo-4-hydroperoxide (*aka* 4-hydroxymethyl-2,6-heptane-dione). The hydroperoxy group (*I-CHAT*-agent) transfers its H atom to the oxygen atom of the carbonyl group and concurrently accepts an H-atom from the CH_2_- group to mediate tautomerization. A similar rearrangement occurs with the second carbonyl group, again, regenerating the catalyst moiety. PES analysis shows that the barriers to classical (direct) H-transfer processes are very high. The first step barrier, for instance, is as high as ΔG^#^(*dir*) = 66.0 kcal/mol, whereas *I-CHAT* catalysis reduces it significantly to as low as ΔG^#^(*I-CHAT*) = 39.64 kcal/mol for the first step, and 39.45 kcal/mol for the second step.

Apparently, the multistep processes can effectively occur when sufficient internal energy is available to overcome the barriers, typically when the molecule is activated chemically or photochemically, as in the case of energized KHP intermediates produced during the combustion of conventional fuels via the dual oxygenation of fuel radicals [3,10,11].

A sequential *I-CHAT* process is modeled for different 2,6-diketo-4XH derivatives of the primary reference fuel heptane, where XH is represented by -CH_2_OH, -COOH, or –SH groups. To explore the possible reactions of alcohol-derivatives, here, we have particularly calculated such a process in case of the CH_2_OH as an *I-CHAT*-agent, calculated at the M06-2X/cc-pVTZ level of theory. Scheme 4 shows a double- *I-CHAT* process catalyzed by OH with the same pentenone backbone in which the hydroperoxy group –OOH in γ-C5-KHP is replaced by the CH_2_OH *I-CHAT* group. Because the TS with only OH as a catalyst in pentane hydroxy-2,4-KHP- could not be located, we added a methylene group to properly extend the TS-rings and reduced the ring-strains, comparable to the OOH group. The results for the CH_2_OH-catalyzed process provided below are basically like those described above.

Notably, the overall energetics changed insignificantly when the hydroperoxy OOH group was replaced by CH_2_OH. This reaction can also be considered as a new type of poly-hydroxide formation reaction, particularly facilitated at elevated temperatures.

**Figure 1:**
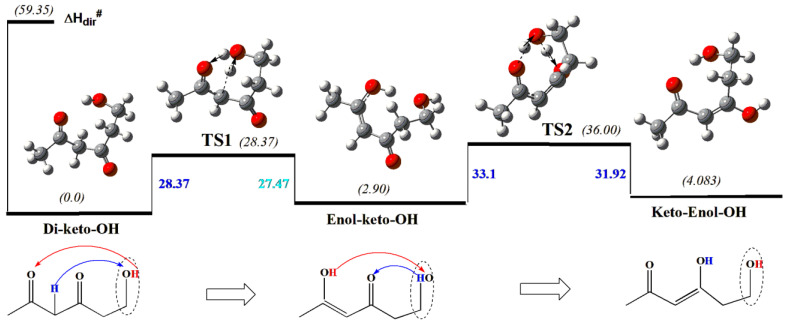
An illustrative example of the more complex long-range and sequential keto–enol tautomerization of a model 2,4-diketo-6-hydroxy hexane—an intermediate in *n*-heptane oxidation. The *I-CHAT* catalyst (OH, or rather encircled CH_2_OH—group) transfers one of its H-atoms to the distant carbonyl oxygen and concomitantly accepts an H-atom from the α-methylene group to produce the 2-enol-4-keto-6-hydroxy hexane product. Another *I-CHAT* rearrangement of the regular type provided in Scheme 2 can occur with the second carbonyl group, again regenerating the catalyst moiety and forming the 2-keto-4-enol-6-hydroxy hexane isomer.

***Long-Range and Combined Models.*** Various longer-range intramolecular catalytic processes may also occur via extended transition states depending on the size and structure of the molecules and dimension and electronic characteristics of the *I-CHAT* groups and the acceptor sites. Figure 1 provides an example of such keto–enol tautomerization involving a model 2,4-diketo-6-hydroxy hexane molecule encountering significantly low sequential barriers of activation. The combustion literature provides a variety of examples of such structures as noted above (e.g., [18,61]). Highly oxidized multifunctional carbonyl compounds are present even in the troposphere [68], with some being identical to those formed during *cool flame* combustion [18].

As one can see, the hydroxymethyl CH_2_OH- group we have “added” to the 2,4-pentane-dione (acetylacetone) to create a relevant model, indeed, can be formally considered as an *I-CHAT*-catalyst group (instead of the actual catalyst OH- group). It is owing to the added extra methylene linkage allowing steric accessibility of OH to the reaction centers – carbonyl groups. Thus, in the first step, the OH- group transfers its H-atom to the distant carbonyl oxygen and accepts an H-atom from the proximal carbon backbone to produce an ***enol–keto–hydroxide*** product through a barrier of 28.4 kcal/mol (Figure 1). Then, the same catalyst OH- group at C6 transfers its H-atom to the remaining proximal carbonyl group, obtaining H- from the newly created enol group (shown by arrows in Figure 1). Both TS rings are six-membered in contrast to the reactions discussed above that involve four and five-membered rings, thus reducing the ring strains and, hence, the corresponding barriers.

We note that a simplified version of the reagent where the internal (central) C=O group is replaced by a simple methylene linkage, also undergoes a similar transformation; however, this is via a substantially higher barrier of 36.54 kcal/mol—almost 8 kcal/mol higher than the original one. This demonstrates the important role of both the electronic and steric factors in determining barrier heights.

Enols can also be responsible for carboxylic acid balance via bimolecular and chemical activation reactions, as suggested by Taatjes and coworkers [79], considering the impact of the *enols* on atmospheric processes, after the discovery of enols as important intermediates in combustion chemistry and flames [76,77,78,79,93]. They suggested that the atmospheric carboxylic acids can be formed not only via the Korcek mechanism but also through reactions with enols (both in the atmosphere and in combustion media).

### 2.3. An Outlook and Possible Implications of the I-CHAT Mechanism

**Figure 2:**
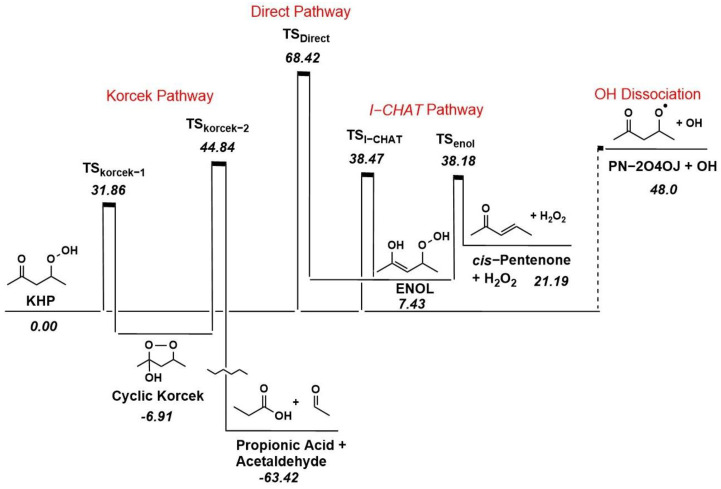
Potential energy diagram for *I-CHAT*, Direct, Korcek, and OH dissociation KHP pathways. Enthalpies (kcal/mol) are relative to the KHP baseline.

The *I-CHAT*-enabled formation of enols opens a new avenue for a variety of processes. Tautomerization can play an important role in the chain-branching events in low and mid-temperature combustion processes because further decomposition of the KHP-derived enols generates various reactive intermediates (reaction 1), among others, H_2_O_2_—a key chain-branching agent in mid and high-temperature combustion. The formation of H_2_O_2_ is facilitated by the proximity of the two hydroxyl groups in the enol form of the γ-KHPs, as shown in Scheme 2 and Figure 2 in Section 2.4. There is some similarity to the Korcek reaction (2), also leading to the unimolecular decomposition of KHPs with a similar energy profile.

KHP → ENOL →  H_2_O_2_, Pentenone, Criegee Intermediate, and others (1)

KHP → Peroxy-Cycle   →  Carboxylic Acids + Carbonyls(2)

However, in contrast to the Korcek pathways, which only produce carboxylic acids and carbonyls, the *I-CHAT*-mechanism can generate a variety of compounds including chain-branching agents (such as H_2_O_2_ and Criegee intermediates), which can alter global combustion characteristics of the fuels (ignition delay times and flame speeds), dissociating more effectively in the higher temperature regime as a consequential reaction (see, e.g., ref. [95]). Some other pathways leading to both species already observed in experiments and new products were also identified for the decomposition of γ-C5-KHP, such as methyl-Criegee Intermediate, vinyl hydroperoxide, acetone, diketone, and others, which will be reported in a separate publication.

Note that H_2_O_2_ has been found to be the most abundant peroxide during the oxidation of *n*-pentane at temperatures below 1000 K (exhibiting two zone behavior—with maxima below and above 800 K) in SVUV-PIMS experiments by Battin-Leclerc and coworkers [65]. Perhaps, such behavior can partly be explained by the operation of the *I-CHAT* mechanism, inviting further exploration.

At intermediate temperatures, the small peroxide species H_2_O_2_ contributes to chain branching through decomposing into two OH radicals [16]. The first reliable quantification of H_2_O_2_ formed during low-T combustion was presented by Bahrini et al. for the oxidation of *n*-butane [96]. Hydrogen peroxide itself is stable up to 1100 K and is typically considered to be formed by H-abstraction reactions of HO_2_ [3]. However, traditional pathways included in the model by Bugler et al. [97] failed to explain experimental results by Rodriguez et al. [65]. Perhaps, H_2_O_2_ can be formed via other channels including also the novel *I-CHAT* pathways for KHP decomposition. Moreover, the new pathway could explain the formation of pentanone products observed in the experiment, but of unclear origin. The pentenone, as the second counterpart of the 2,4-enol decomposition generating H_2_O_2_ (Table 2), has been identified during the low-T oxidation of *n*-pentane by GC-MS experiments with the onset of detection at 590 K [66].

The simplified version of the combined *I-CHAT* reaction described in Figure 1 can also be considered as a new type of poly-hydroxide formation reaction, particularly facilitated at elevated temperatures. Perhaps enzymes also could employ similar pathways via lower energy ionic versions supported by solvation and tunneling effects.

We also note that more stable *I-CHAT* catalytic systems, such as the substituted di-ketone hydroxymethyl, containing a more robust CH_2_OH group instead of a hydroperoxyl group with a weaker O–OH bond described in Scheme 2, can allow the *I-CHAT* mechanism to operate at much higher temperatures relevant to flames.

The reactions of enols contribute significantly towards atmospheric carboxylic acid concentrations in the gas phase, as noted above [41,75]. The *I-CHAT* pathways could further fortify this conclusion.

We thus suggest that there should be many other processes facilitated by *I-CHAT* pathways. The novel mechanism may have important ramifications in a variety of processes, including waste oil degradation and aerosol formation and degradation.

It is important to note that experimentally distinguishing between the direct and *I-CHAT* intramolecular mechanisms is challenging compared to distinguishing between direct vs. bimolecular (intermolecular catalysis) processes. To isolate a direct *unimolecular* keto–enol tautomerization pathway from alternative *bimolecular* molecular-catalysis processes involving an extra-molecule as a catalyst, such as water and carboxylic acids typically dilute conditions are used, as stressed by Labbe and coworkers [83], such that the chemistry is not clouded by subsequent bimolecular chemistry. On the other hand, the emerging additional unimolecular pathway (*I-CHAT)*, which would not be altered by diluting, can certainly complicate the diagnostics of the classical reactions and even cast some doubt on the ambiguity of the experimental identification of bimolecular *molecular-catalysis* processes when *I-CHAT* channels are available.

Thus, designing experiments to distinguish two alternative intramolecular pathways—an *I-CHAT* pathway vs. an uncatalyzed (direct) pathway—will be quite challenging. Perhaps, indirect evaluation of secondary reactions or isotopic labeling could be of help, albeit in both cases the labile O–H and “unwastable” C–H skeletal bonds are combined similarly in the reaction schemes, whereas the OOH- group is involved only in the *I-CHAT* mechanism, as highlighted in Scheme 2. The exploration of coupled PES could shed light since the results can differ significantly from those calculated on separate PESs. Part of the “hot” KHPs could certainly dissociate via new channels.

### 2.4. Kinetic Analysis of an I-CHAT Process Employed for Model Generation

A set of thermochemical properties was calculated for KHP and important species pertaining to our *I-CHAT* mechanism, classic (direct) keto–enol tautomerization, oxy, and hydroxyl radical formation via direct dissociation and Korcek pathways using the M06-2X/aug-cc-pVTZ method. High-pressure rate constants were calculated using the Automated Reaction Kinetics and Network Exploration (Arkane) [98] program as distributed in the Reaction Mechanism Generator-Py (RMG v3.2.0) program [99,100,101]. The rate coefficients were calculated using transition state theory and fitted to the three-parameter form of the Arrhenius equation. Temperature and pressure-dependent rate coefficients were calculated using the Rice–Ramsperger–Kassel–Marcus (RRKM) theory to solve the master equation with the modified strong collision approximation.

Substituted hydroperoxide groups, such as KHP, predominately dissociate to generate oxy and hydroxyl radicals due to the relatively weak oxygen–oxygen bonds [43], to participate in subsequent chain-branching reactions. Our *I-CHAT*-enabled formation of enols should be able to compete at lower temperatures along with propionic acid and acetaldehyde formation via the Korcek decomposition.

To show the relative barrier heights to some comparable KHP pathways, a potential energy diagram is shown in Figure 2 for γ-C5-KHP (here, KHP for simplicity). As discussed above in Scheme 2, the barrier height of the *I-CHAT* pathway to create the enol-hydroperoxide ENOL is approximately 39 kcal/mol higher relative to KHP, while the classic (direct) keto–enol tautomerization of KHP is 30 kcal/mol higher than this at 68 kcal/mol. A breakdown pathway for ENOL is then possible via hydrogen peroxide loss over a 31 kcal/mol TSenol barrier, where a hydrogen transfer from the newly created hydroxyl group to the hydroperoxide group generates *cis*-pentenone. KHP has also been shown to form a cyclic peroxide followed by subsequent carbonyl and carboxylic acid products via the Korcek mechanism with barriers of 32 and 52 kcal/mol for TSkorcek-1 and TSkorcek-2. For comparison, Jalan et al. [27] determined barriers of approximately 35 and 29–32 kcal/mol for cyclic peroxide and subsequent product formation for the three-carbon ketohydroperoxide. The dissociation limit for KHP is 48 kcal/mol for oxygen–oxygen bond breaking forming hydroxyl and oxy radical.

**Figure 3:**
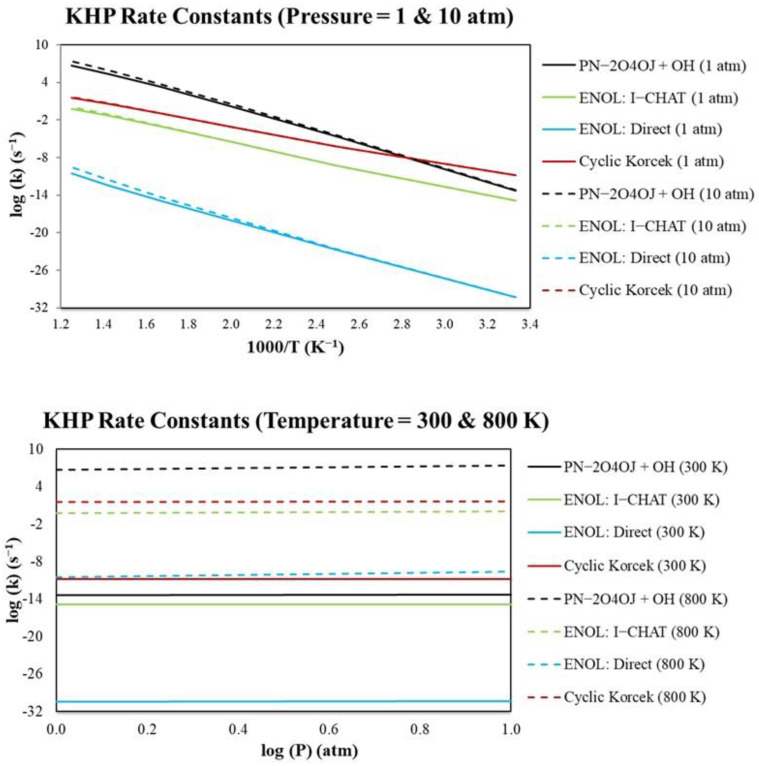
Rate constants vs. pressure (1 and 10 atm) and vs. temperature (300 and 800 K) for KHP pathways.

Calculated rate constants for the species in Figure 2 are summarized in Table 2 below, and sample pressure and temperature values are included in Figure 3. The temperatures and pressures in Figure 3 provide a larger range to foundationally gauge the representative nature of our *I-CHAT* pathway and how it can compare to two other important known oxidation decomposition pathways. Overall, in the top portion of Figure 3 for 1 and 10 atm pressures, the Korcek pathway is favored from 300 K to approximately 350 K, at which point the dissociation overtakes it, while the *I-CHAT* is consistently lower across all the temperatures up to 800 K. As the temperature increases, Korcek and *I-CHAT* begin to converge together albeit away from dissociation simultaneously. Also, increasing pressure produces a slight increase in the rate constant above approximately 600 K and is more pronounced for the dissociation and *I-CHAT* pathways. Comparatively, *I-CHAT* is significantly faster than the direct (classical) enol formation pathway. In the bottom portion of Figure 3, rate constants are shown from 1 to 10 atm at temperatures of 300 and 800 K. Rate constants are almost constant throughout this region, with the direct enol formation pathway being the lowest. At 300 K, Korcek is favored; meanwhile, at 800 K, dissociation occurs faster. At both temperatures, the *I-CHAT* mechanism is comparable to dissociation at 300 K, and, for Korcek, it is at 800 K.

In this initial overview comparison of our *I-CHAT* mechanism to two important oxidation pathways and the classical direct enol formation, at the temperatures and pressures considered for KHP, *I-CHAT* has the potential to be an important pathway to consider. A future, more expansive analysis will serve as a basis to put our *I-CHAT* pathway in greater perspective to see its potential impact on the low-temperature oxidation mechanism for hydroperoxide chemistry and mid- to higher-temperature processes via other more stable compounds, such as multi hydroxy-aldehydes (ketones), described in Scheme 4 and Figure 1.

We again emphasize that the *I-CHAT* mechanism generates species that can serve as chain-branching agents, such as H_2_O_2_ provided in Figure 2, and Criegee, to alter global fuel characteristics, such as IDT and flame speeds, as opposed to the Korcek pathway, which forms only acids and carbonyls as primary products, leading to chain termination and the removal of KHP. Thus, even a relatively small flux through the I-CHAT mechanism could have a non-trivial impact on ignition.

### 2.5. Chemical Kinetic Model Generation Using RMG and Simulation of IDT

To explore the impact of the new reaction pathways on the combustion of large alkanes, we have generated a set of chemical kinetic models for the oxidation of *n*-pentane, the smallest relevant model of the large alkanes, using Reaction Mechanism Generation (RMG v3.2.0) software [99,100,101]. The simulation of their performance for predicting ignition delay times (IDTs) at given conditions was performed using CHEMKIN [102,103]. The main mechanism, denoted here as ***PN-ML-m-v1*** and reported earlier [21], was compared with newly generated alternative mechanisms, particularly the one supplemented with *I-CHAT* reaction channels (***PN-ML-m-v2***).

RMG constructs kinetic models using libraries of known reactions and kinetics, generalized chemical knowledge, rate rules, and group additivity (GA) techniques [99,100,101]. It uses a set of “reaction families” to generate all possible reactions that a given chemical species can undergo in the presence of other species in the mechanism. Each reaction family represents a particular type of elementary reaction, such as radical recombination or addition to a double bond. There are currently 74 reaction families defined in RMG [99,100,101], with three new ones added in this work described below. RMG employs a rate-based algorithm to determine which species and reactions are included in the model. Species and their associated reactions are added iteratively until the production rates of all species drop below a specified termination threshold. Thermochemical data for species and reaction rate parameters are sourced from a hierarchy, beginning with experimental values in libraries, and utilizing GA and rate rules when experimental data and fundamental calculation results are unavailable. RMG is one of the most advanced and widely used software tools in the field, featuring a stable and robust architecture for developing extensible, modular code.

In this study, RMG v3.2.0 was utilized as the primary tool to generate detailed kinetic models. A simple reactor model that is relevant to the gas phase processes was further employed.

To test the model predictions (our simulation results), we used experimental data from the National University of Ireland, Galway (NUIG), for the combustion of pentane isomers, as well as the manually constructed model of the NUIG as a benchmark [104]. The latter model has been tested and validated against rapid compression machine (RCM) and shock tube (ST) data from NUIG [104].

To incorporate *I-CHAT* pathways in the model generations, we have developed three new reaction families viz. **2,4**-**1,3*_CHAT*** and **2,5_*CHAT*** for catalyzed keto–enol conversions of 2,4-, 1,3-, and 2,5- KHPs via *I-CHAT*, as well as the ***ENOL*** family to describe the dissociation of 1,3*-* and 2,4- enol products to form 2*-pentenal*, *cis-pentenone*, and H_2_O_2_ (Table 1), and incorporated them into RMG. To add these new pathways to the RMG family database, we used the Subgraph Isomorphic Decision Tree (SIDT) algorithm [105]. This method is particularly effective when dealing with smaller datasets where traditional machine learning models, such as neural networks, fall short. Rather than requiring thousands of reactions to learn effectively, SIDT works by breaking down reactions into recognizable patterns through subgraph matching. As the decision tree evolves, each step refines these patterns, making the predictions increasingly accurate and easier to interpret. The process begins with the root node, which represents the initial, broadest reaction template for the reaction family under study. This root node is defined by a general structure that captures the fundamental features of the reactions, using placeholders like generic groups or unspecified bonds to ensure flexibility. The root node should be broad enough to encompass a wide range of reactions within the dataset. For example, to generalize I-CHAT reaction channels for larger molecules, we generated a generic root capable of expanding to molecules as large as C_32_H_66_.

Following the procedures described in [105], we created a root for these new families and, by providing training reaction parameters calculated in the kinetic part of this study (Section 2.4), as listed in Table 3, we calculated branches based on a decision tree to be generalized for similar cases.

The rate parameters employed to parameterize these new reaction families were those described in the previous section. The fundamentally based thermochemical data, included in a separate RMG library, were provided for eight added species included in the new families. The calculation of the thermochemical properties for those species is based on the M06-2X/aug-cc-pVTZ method using the ARM-2 atomization approach [106] to be consistent with the calculated rate parameters described above.

To evaluate the ignition delay times (IDT), we used the Chemkin Pro 2023 model provided by ANSYS [102,103]. Calculations were performed for a homogeneous constant-pressure batch reactor, where the ignition delay time was defined as the time required for the temperature to increase by 400 K. In generating the *n*-pentane oxidation mechanism, our approach prioritized experimentally verified and first-principles-based data sources as much as possible, while relying on rate-based criteria to expand the mechanism.

**Figure 4:**
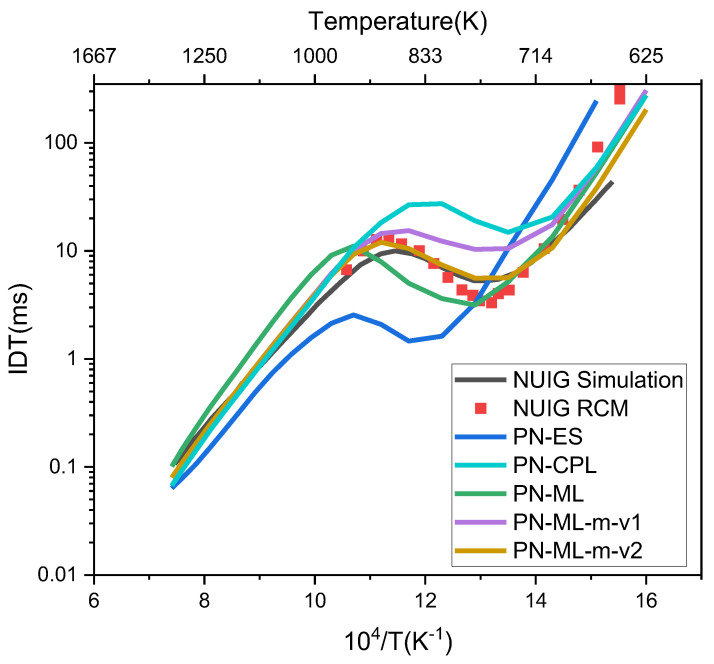
Effect of ***I-CHAT*** reactions (new kinetic families and thermochemical data) on PN-kinetic model performance for *ϕ* = 2 and *P* = 10 atm conditions. Models were generated using: (a) no libraries, only estimation methods (***PN-ES***), (b) only the Curran Pentane as a Library (***PN-CPL***), (c) higher fidelity data via multiple libraries (***PN-ML***), (d) using multiple libraries supplemented with our modified reaction library (***PM-ML-m-v1***) from our previous study [21], and (e) multiple libraries and our modified reaction library supplemented with three new kinetic families for the *I-CHAT* mechanism and new thermochemistry data (***PN-ML-m-v2***). Symbols represent experimental data; lines are model predictions.

Figure 4 shows the IDT performance for a wide range of temperatures from *T* = 625 K to 1350 K at *P* = 10 atm and fuel-rich conditions (*ϕ* = 2); *ϕ* is the equivalence ratio of fuel to oxidizer. The simulation results for fuel-lean and stoichiometric conditions are provided in Supplementary Materials.

Overall, we generated five different models. In addition to those described above (***PN-ML-m-v1*** and ***PN-ML-m-v2***), a simpler model was generated (“from the scratch”) based only on *estimation* methods, rate rules for reaction kinetics, and group additivity for thermochemistry provided in RMG, denoted as ***PN-ES.*** Despite reproducing the NTC behavior, the predicted IDTs are too low and shifted to higher temperatures compared to experimental data for this selected condition, as seen in Figure 4. In another case, to further support pentane combustion modeling, we included only the manually developed NUIG mechanism by Bugler et al. [104], implemented in RMG as the CurranPentane Library (CPL). CPL provides a whole range of thermochemical and kinetic parameters for pentane combustion. CPL in RMG mainly serves as a source for both thermochemistry (primarily for C5 and some C4 species) and reaction kinetics for pentane isomers. It is partly based on high-fidelity experimental and fundamental data and is partly estimated and tuned to their experiments [104]. It should be emphasized that the complete NUIG mechanism (comprising 675 species and 3065 reactions) is too large to serve as a basic seed mechanism for generating combustion and pyrolysis models for larger paraffins while maintaining a manageable mechanism size. Therefore, we employed CPL as a library to provide RMG with data derived from the NUIG mechanism [104]. As can be seen from Figure 4, the performance of ***PN-CPL*** is better than that from purely estimation methods (***PN-ES***); however, the system remains less reactive for the negative temperature coefficient region, compared with experimental data and the full NUIG model predictions.

Furthermore, we utilized a set of other reaction and thermochemistry libraries available in RMG, primarily focusing on high-fidelity data for small-species chemistry, denoted as ***PN-ML*** (multiple libraries). For the H_2_/O_2_ reactions, we used the Burke sub-model for H_2_ combustion at high pressures [107]. As a source of C1–C2 chemistry, we used the Klippenstein–Glarborg model library, derived from the recent mechanism published by Hashemi et al. [108]. Additionally, thermochemistry data for C3–C4 species were provided using updated databases available in RMG. Specifically, the thermochemistry was sourced from a database by Goldsmith et al., which includes refined QCISD(T) energies for numerous combustion-relevant small species (“DFT_QCI_thermo”), along with composite-level predictions from CBS-QB3 (“CBS_QB3_1dHR”) and G4MP2 methods [109]. To improve ***PN-ML*** model performance, we have previously created and applied a *modified* reaction and thermochemistry library described in detail in ref. [21]. The mechanism denoted here (***PN-ML-m-v1***) represents the first (basic) version of the modified ***PN-ML-m*** model.

The final version of this mechanism (***PN-ML-m-v2***) additionally involves all three new families and trained reactions from Table 1.

As can be seen in Figure 4, adding the modified library in ***PM-ML-m-v1*** improved the model’s performance at the defined conditions. Here, high-pressure and fuel-rich conditions are most relevant to the engine and HRF combustion (see also Supplementary Materials). The performance of both versions of the ***PN-ML-m*** model is significantly improved compared to the original ***PN-ML*** model, where we only relied on the databases from RMG, as well as ***PN-CPL*** using only the CPL database. Further addition of catalytic *I-CHAT* isomerization and ***ENOL*** dissociation pathways via new families (***PN-ML-m-v2***) improves the model predictions even more.

To conclude, the novel *I-CHAT* pathways, along with enol dissociation reactions, are important new pathways to be added to alkane combustion mechanisms to significantly affect the model performance. It is particularly due to the formation of chain-branching H_2_O_2_ as the most abundant peroxide in low-T combustion of *n*-pentane, as shown by Bourgalais et al. by coupling a jet-stirred reactor with an electron/ion coincidence spectrometer, not fully explained through traditional pathways [66].

## 3. Methodological Details

A detailed potential energy surface (PES) analysis of reaction pathways was performed using the generalized-gradient approximation (GGA) M06-2X hybrid density functional theory from the Truhlar (Minnesota) group [110], in conjunction with Dunning’s correlation consistent aug-cc-pVTZ basis set [111]. Smaller basis sets were employed for detailed screening of the PES for *I-CHAT* rearrangements and classical H-transfer reaction barriers. The M06-2X method is well-tested in the literature in the same domain, including our previous studies [112,113,114,115,116]. It has been particularly recommended for tautomerization processes by Acevedo and coworkers in predicting experimental gas-phase free energies for various keto–enol tautomerization processes, including the γ-diketo pentane to 2-keto-4-hydoxy-3-pentene [82], which is closely related to the processes studies here. For benchmarking the results, we have also performed selected single-point CCSD(T)/6-311+(2d,p) calculations for key models to validate the DFT results. For larger models and initial PES analyses, a moderate 6-31+G(d,p) Pople-type basis set augmented with diffuse and polarization functions was employed as recommended by Truhlar et al. as the best affordable basis set for the exploration of reaction barriers in large molecular systems [117]. All results presented here are at the M06-2X/aug-cc-pVTZ level unless otherwise stated.

The first-order saddle points were characterized as having only one negative eigenvalue of their Hessian matrices. The absence of imaginary frequencies verified that reactant and product structures were true minima at their respective levels of theory. The intrinsic reaction coordinate (IRC) analysis was performed to ensure proper connectivity of stationary points. All PES calculations were performed using the Gaussian 16 program (revision A.03) [118].

High-pressure-limit rate constants, k(T), were calculated for the 300–2000 K temperature range using the automated reaction kinetics and network exploration software (Arkane) code [98] as part of the Reaction Mechanism Generator (RMG v3.2.0) program complex [99,100,101]. The rate constants utilize transition state theory with the reaction path symmetry number, energy barrier, and the reactant and transition state well partition functions with tunneling corrects from the Eckart correction. Temperature and pressure-dependent rate constants, k(T,P), were also calculated in Arkane using the Rice–Ramsperger–Kassel–Marcus (RRKM) theory with the modified strong collision method.

The calculated rate parameters were incorporated into an *n*-pentane kinetic model developed earlier using the RMG automated model generation tool [21,81] to evaluate the impact of the new reactions on ignition (global fuel combustion) characteristics of *n*-pentane.

The simulation of the IDT was performed by Chemkin [102] software using ANSYS Chemkin-Pro 2023 [103] to carry out ignition delay calculations for a homogeneous constant-pressure batch reactor, taking the ignition delay time to be the time required for the temperature to increase by 400 K.

## 4. Summary and Conclusions

A novel unimolecular decomposition mechanism of ketohydroperoxides involving “internally catalyzed transfer of hydrogen atoms” (*I-CHAT*) has been developed and applied to the combustion of large alkanes. Low energy pathways were identified for keto–enol tautomerization of the model chain branching intermediates ranging from skeletal models to larger alkanes, *n*-pentane, *n*-hexane, and *n*-heptane, serving as prototypes for gasoline, diesel, and hybrid rocket fuels. The newly formed γ-C5-EHP enol-hydroperoxide isomer of pentane-derived ketohydroperoxide (γ-C5-KHP) was shown to easily eliminate another chain branching agent, H_2_O_2_, and to form the experimentally observed pentenone co-product.

The rate parameters for these processes were calculated and shown to compete with two main, alternative KHP decomposition channels—direct dissociation of OH- radicals (main chain branching event in the traditional mechanism of fuel ignition), and Korcek decomposition to form acid and a carbonyl compound. The implementation of rate parameters in a kinetic model developed previously using RMG automated reaction mechanism generation tools to examine its role in the prediction of global combustion characteristics was presented.

Overall, we find that the O–O bond fission remains the dominant process of KHP decomposition due to the higher entropy gain despite the noticeably higher O–O bond-breaking energy of γ-KHP compared to the alternative transition state barriers for the novel *I-CHAT*-tautomerization and the Korcek reactions. The keto–enol pathway somewhat resembles the Korcek mechanism in terms of the formation and decomposition of an isomer of KHP; yet, it generates more diverse products, including other chain-branching and chain-propagation agents H_2_O_2_ and Criegee Intermediates. The formation of γ-EHP in the simple case of the γ-KHP encounters an activation barrier somewhat higher than that for the first step Korcek reaction (38.5 vs. 31.9 kcal/mol); however, its further decomposition versus the similar second Korcek step is evidently more viable (38.2 vs. 44.8 kcal/mol).

Longer-range and sequential *I-CHAT* processes were also shown to occur depending on the dimension and electronic characteristics of the *I-CHAT* group and the proton affinity of the acceptor sites.

Simulation of ignition delay times based on RMG-generated models with and without including new pathways compared with experimental data showed the significant effect of the *I-CHAT* pathways on the low-temperature combustion of traditional fuels.

The generation of new chain branching and propagation agents facilitated by the *I-CHAT* mechanism and its possible heterogeneous analogs may have important ramifications in combustion chemistry, waste oil degradation, and other processes involving peroxy chemistry. They might also be important in chemical oscillation reactions (e.g., the conversions of malonic acid [119]) and autocatalysis reactions (e.g., acid-catalyzed hydrolysis of esters and formose reaction involving catalyzed H-migration processes [120]), as well as atmospheric (photochemically activated) aerosol formation and degradation [121,122,123].

## Figures and Tables

**Table 2:** Fitted, modified Arrhenius equation parameters for high-pressure rate constants for *I-CHAT*, Direct, Korcek, and OH dissociation KHP pathways.

Reaction Pathway	A (s^−1^)	n	E (kcal/mol)
KHP → Cyclic Korcek	8.46 × 10^−3^	3.70	24.99
Cyclic Korcek → Propionic Acid + Acetaldehyde	1.89 × 10^−10^	1.17	51.50
KHP → ENOL (*I-CHAT*)	2.37 × 10^−5^	4.57	30.06
KHP → ENOL (Direct)	8.86 × 10^−42^	15.74	40.02
ENOL → *cis*-Pentenone + H_2_O_2_	5.53 × 10^8^	1.20	29.64
KHP → PN-2O4OJ + OH	4.33 × 10^19^	0.80	47.80

**Table 3:** Rate constants for *I-CHAT* and Enol decomposition training reactions (Section 2.4) employed to create a decision tree for three new reaction families.

Reactions	A (s^−1^)	n	E (kJ/mol)
KETO_24 = ENOL_24	2.37 × 10^−5^	4.57	125.8
KETO_13 = ENOL_13	416	2.53	137.8
KETO_25 = ENOL_25	8.42 × 10^12^	6.53	111.2
ENOL_13 = 2-Pentenal + H_2_O_2_	1.194 × 10^11^	0.482	115.9
ENOL_24 = H_2_O_2_ + *cis*-Pentenone	5.531 × 10^8^	1.199	124.0

## Data Availability

Data are contained within the article and Supplementary Materials.

## Supplementary Materials

The following supporting information can be downloaded at: https://www.mdpi.com/article/10.3390/molecules30030524/s1, Optimized Structures and Calculated Energies, Simulation Results, and Complete References.
