# Supplementary material for: Internally Catalyzed Hydrogen Atom Transfer (I-CHAT)—A New Class of Reactions in Combustion Chemistry
Source: Molecules. 2025 Jan 24;30(3):524. doi: 10.3390/molecules30030524 (PMC11820802; doi:10.3390/molecules30030524)
Supplement: Supplementary file 1 [file molecules-30-00524-s001.zip › molecules-3429514-supplementary.pdf]

SUPPORTING INFORMATION

Internally Catalyzed Hydrogen Atom Transfer (*I-CHAT*)—A New Class of Reactions in Combustion Chemistry

Department of Chemical and Biological Engineering and Center for Hybrid Rocket Exascale Simulation Technology (CHREST), University at Buffalo, The State University of New York, Buffalo, NY 14260, USA;

Section	Page
Optimized Structures and Calculated Energies	S2-S28
Simulation Results	S29-S30
Complete References	S31

Optimized Structures and Calculated Energies

Optimized structures and some calculated energies from the Schemes/Tables/Figures in the manuscript as indicated from the M06-2X/aug-cc-pVTZ (unless otherwise noted) level of theory.

Optimized Structures from Scheme S2

Scheme S2. Internally catalyzed hydrogen atom transfer (*I-CHAT*) mechanism versus *Direct* keto-enol tautomerization of the model pentane-γ-ketohydroperoxide (γ-KHP) - a key chain-branching agent of the combustion of traditional fuels. Relative enthalpies are provided in parenthesis. *I-CHAT* reduces the barrier by as much as 22 kcal/mol.

Keto-Hydroperoxide (KHP)

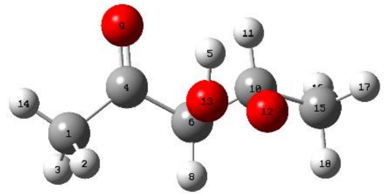

C	0.71873400	8.80294600	5.15037200
H	0.66187600	9.81591500	4.76397900
H	1.74612800	8.43726300	5.12324800
C	-0.17464500	7.88725900	4.36103300
H	-1.47434000	6.73613600	2.38528500
C	-0.11400800	6.40468200	4.66101300
H	-1.13741100	6.03660400	4.70799300
H	0.37090000	6.21629600	5.61864500
O	-0.92720700	8.30978000	3.51284500
C	0.63944500	5.62252000	3.56790700
H	0.42377400	4.56059300	3.71655300
O	0.22814700	5.99863400	2.26787300
O	-1.17667300	5.83640000	2.16211500
H	0.40528400	8.78574000	6.19633700
C	2.13697400	5.86263400	3.57416700
H	2.56016700	5.59242900	4.54066700
H	2.61359400	5.26148600	2.80263900
H	2.35693500	6.91054900	3.36918200

Sum of electronic and zero-point Energies=	-421.969360
Sum of electronic and thermal Energies=	-421.960184
Sum of electronic and thermal Enthalpies=	-421.959240
Sum of electronic and thermal Free Energies=	-422.003180

$TS_{I\text{-}CHAT}$

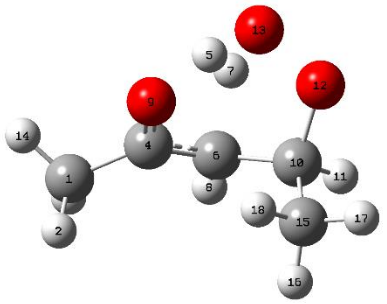

υ1=- 1704.3133

C	0.35956200	8.90046200	5.41337300
H	1.35019400	9.32890500	5.24745700
H	0.32183400	8.50820700	6.42617700
C	0.15269800	7.82408300	4.39478100
H	-0.86107500	7.16774800	2.79982200
C	0.24256800	6.45238700	4.72471500
H	-1.04015400	6.22806000	4.09239500
H	0.43174700	6.20232600	5.75766400
O	-0.19668500	8.18217700	3.22531300
C	0.77020800	5.46982700	3.69731200
H	0.85967800	4.49424600	4.17741500
O	-0.21759300	5.30914300	2.62152600
O	-1.33895700	6.12411000	2.92783500
H	-0.37308300	9.69263400	5.27769800
C	2.07732600	5.84490800	3.02661700
H	2.88339200	5.83683600	3.75991600
H	2.31427500	5.12701400	2.24188900
H	2.00378600	6.83593100	2.58162200

Sum of electronic and zero-point Energies=	-421.906572
Sum of electronic and thermal Energies=	-421.898254
Sum of electronic and thermal Enthalpies=	-421.897310
Sum of electronic and thermal Free Energies=	-421.939763

TS$_{dir}$

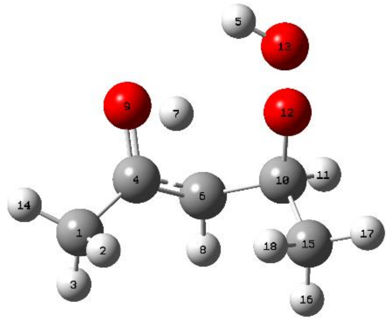

C	0.66546300	8.93102200	5.32676300
H	1.53760900	9.15651800	4.70792100
H	1.03685200	8.60215100	6.29576900
C	-0.08117600	7.82572500	4.67504600
H	-1.65889500	6.62167400	2.08879000
C	0.10534500	6.42252600	4.75251100
H	-1.20040000	6.84789600	4.16357900
H	0.55728300	6.02271700	5.65582300
O	-1.11342500	8.07128000	3.94964800
C	0.63890700	5.68049800	3.51330800
H	0.36253700	4.62875700	3.61381100
O	0.13836400	6.15720300	2.27640200
O	-1.22166400	5.76199400	2.14494600
H	0.05745200	9.82495500	5.43130100
C	2.14766100	5.80982400	3.35995700
H	2.63982800	5.47670700	4.27176300
H	2.50164800	5.20881000	2.52351000
H	2.41593300	6.85172800	3.18162000

Sum of electronic and zero-point Energies=	-421.859997
Sum of electronic and thermal Energies=	-421.850525
Sum of electronic and thermal Enthalpies=	-421.849581
Sum of electronic and thermal Free Energies=	-421.894607

Enol-Hydroperoxide

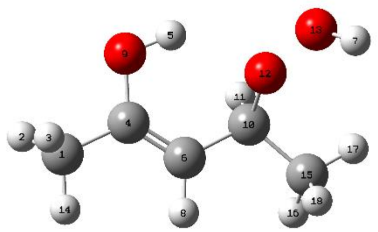

C	0.19968700	9.00493900	5.46597500
H	-0.76651500	9.50215900	5.37707700
H	0.96924100	9.75528000	5.28227700
C	0.31137900	7.91931900	4.44916500
H	0.16397600	7.67594500	2.55587400
C	0.51925900	6.63018200	4.73398700
H	-1.64582800	6.45595700	3.47707900
H	0.60514700	6.35205000	5.77426800
O	0.16360500	8.40926200	3.18940700
C	0.68177700	5.54232900	3.70593900
H	0.37919900	4.58270900	4.12902000
O	-0.14261900	5.78006100	2.55555400
O	-1.50271900	5.66489100	2.93412800
H	0.31340500	8.61239200	6.47233000
C	2.09512800	5.45376400	3.15284100
H	2.78613000	5.23802900	3.96548900
H	2.16610700	4.66693000	2.40379900
H	2.38543100	6.40453800	2.70546700

Sum of electronic and zero-point Energies=	-421.957108
Sum of electronic and thermal Energies=	-421.947724
Sum of electronic and thermal Enthalpies=	-421.946779
Sum of electronic and thermal Free Energies=	-421.991089

Optimized Structures from Table S1

Table 1. Intramolecular *I-CHAT* - Catalyzed Tautomerization of Truncated Tri-Carbon Models.

A=CH-CH$_2$-CH$_2$-XH

A = **O;** XH = **OOH**

REAGENT _C3_m062xVTZ_KETO

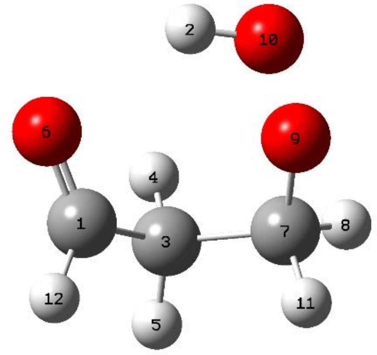

C	0.15385400	7.86641800	4.38290800
H	-1.34917900	6.70518900	2.53971100
C	0.07606300	6.43360300	4.82985400
H	-0.97135800	6.16320600	4.94577300
H	0.59621800	6.29277700	5.77697400
O	-0.69257400	8.38033600	3.69893800
C	0.72085400	5.53554400	3.76426100
H	0.47202400	4.49198600	3.96673500
O	0.33373500	5.89186800	2.45685500
O	-1.07995500	5.78625800	2.37390000
H	1.80570500	5.64834700	3.75119100
H	1.05283500	8.44004100	4.67116900

PRODUCT _C3_m062xVTZ_ENOL

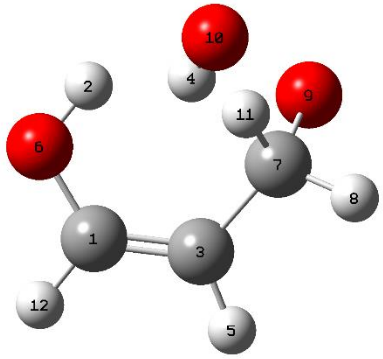

C	0.24657600	7.81196200	4.57114400
H	-0.18628500	7.68165600	2.72339700
C	0.52737200	6.53080200	4.80797600
H	-1.91679600	6.13724500	3.41235000
H	0.66234800	6.23144100	5.83675300
O	0.00477100	8.37501300	3.37003100
C	0.69550000	5.50941800	3.72782500
H	1.29872300	4.67077100	4.07215200
O	-0.51970900	4.88671300	3.31301100
O	-1.31091600	5.89500500	2.69884200
H	1.17817200	5.93713200	2.84383100
H	0.19635500	8.54098400	5.36958700

TSI-CHAT_C3_VTZ

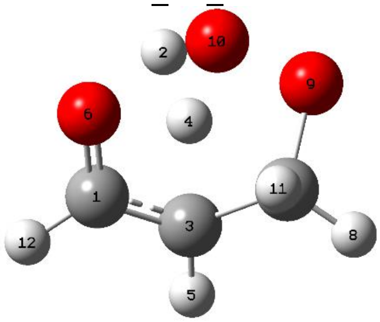

C	0.20074300	7.79953200	4.41602900
H	-0.75194800	7.09316700	2.72567400
C	0.24615600	6.43867600	4.76748900
H	-1.07193500	6.31490900	4.07005400
H	0.36289800	6.17031400	5.80523300
O	-0.00386700	8.17233400	3.22908100
C	0.74897200	5.49942900	3.70477100
H	1.09200900	4.55454200	4.11646600
O	-0.33699900	5.14534100	2.78350800
O	-1.30902300	6.17235200	2.91991500
H	1.52988900	5.95828600	3.09607500
H	0.24738600	8.56943300	5.19279300

A = **O; XH = CH$_2$OH**

REAGENT _C3=O_CH2OH_VTZ

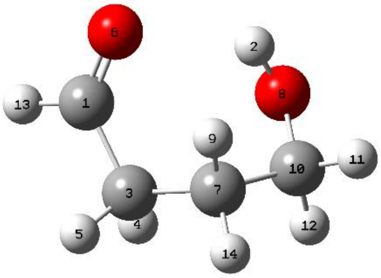

C	2.95059100	5.89367800	7.27525800
H	4.83660900	5.76303400	5.41312200
C	2.03500200	5.54868300	6.13383900
H	1.73006900	6.49311100	5.67100700
H	1.12841200	5.14014000	6.59147400
O	4.05656400	5.44755700	7.43198500
C	2.63501600	4.59092500	5.11370000
O	4.43041000	6.19197700	4.65223800
H	3.23673100	3.84592000	5.63807900
C	3.49182100	5.30652300	4.07761900
H	3.98923200	4.56888600	3.44012000
H	2.85755100	5.92140300	3.43613600
H	2.53142500	6.59820900	8.01685800
H	1.84132500	4.05418600	4.59298700

TSI-CHAT _C3=O_CH2OH_VTZ

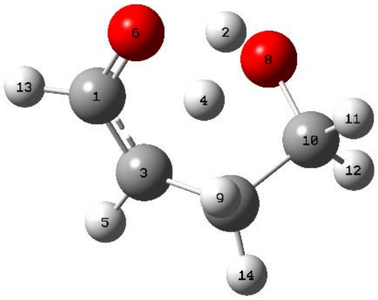

C	3.00603100	5.77282500	7.21705200
H	4.38725700	6.07980400	5.67309100
C	2.09550900	5.52594500	6.18108000
H	2.93038100	6.54719900	5.42422200
H	1.04723500	5.68274400	6.38360500
O	4.25589900	5.65213700	7.08162700
C	2.50785400	4.52823700	5.10531900
O	3.91881300	6.42713700	4.78907600
H	3.03678600	3.69161700	5.56316500
C	3.45544200	5.23913100	4.11144800
H	4.31530800	4.63533600	3.83799200
H	2.94563100	5.58112900	3.21360100
H	2.63029700	6.18652400	8.16080100
H	1.63672600	4.13558900	4.58678800

PRODUCT _C3=O_CH2OH_VTZ

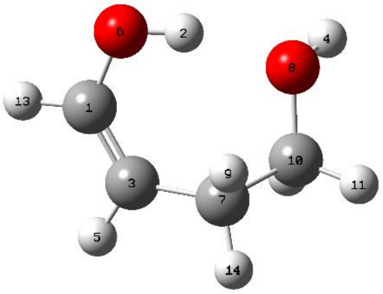

C	2.62271800	5.81967700	7.32237900
H	4.36508700	5.89492000	6.56657300
C	1.98318300	5.19827900	6.33427100
H	4.66447900	6.77802200	4.16037500
H	0.90415000	5.17792900	6.39317300
O	3.95829400	5.98192500	7.44312700
C	2.64903700	4.48932400	5.18494600
O	4.39114700	6.03113200	4.69594400
H	3.45986900	3.85197300	5.55211700
C	3.24144100	5.40450400	4.12683100
H	3.53891000	4.82640800	3.24801100
H	2.51023700	6.15820500	3.82579800
H	2.08468800	6.24738200	8.15874800
H	1.92886800	3.83048200	4.70041400

A = **O;** XH = **COOH**

REAGENT _C3=O_HO(O=)C_VTZ

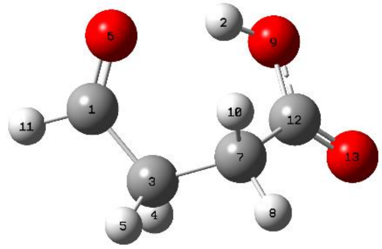

C	-0.04158300	7.78158100	4.53437200
H	-0.57411600	6.91864400	2.20506800
C	0.25719700	6.34379500	4.84023200
H	-0.63221800	5.91463300	5.31315400
H	1.02218700	6.34640600	5.62175700
O	-0.03746400	8.25776900	3.42722900
C	0.71902900	5.51276000	3.64332600
H	1.18951900	4.59913100	3.99335300
O	-0.98227700	6.06070200	2.00232100
H	1.44810500	6.08430800	3.06654900
H	-0.26607300	8.42052200	5.40496800
C	-0.42496800	5.08972000	2.73676700
O	-0.82414400	3.96326900	2.67991200

PRODUCT _C3=O_HO(O=)C_VTZ

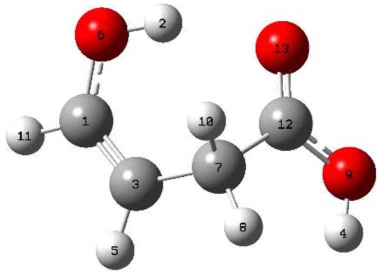

C	0.45721600	8.00239600	4.19683200
H	0.35383300	7.58184000	2.32803900
C	0.36903600	6.77745300	4.71377500
H	-1.50257500	4.14871400	4.03155100
H	0.22704500	6.68705500	5.77941200
O	0.61490200	8.32282400	2.90214100
C	0.50396200	5.51764000	3.88994900
H	0.54927600	4.64302200	4.53652300
O	-1.65020200	4.59132500	3.19057500
H	1.42328700	5.54618800	3.30137200
H	0.42559800	8.87717300	4.83354500
C	-0.63281200	5.41267200	2.89586000
O	-0.66402300	6.06201000	1.88466900

TSI-CHAT _C3=O_HO(O=)C_VTZ

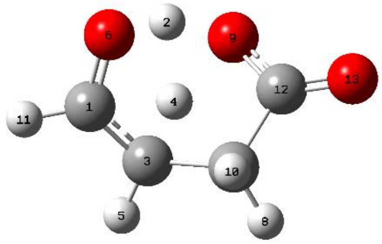

C	0.13748800	7.80319800	4.36997500
H	-0.70423800	7.32113600	2.78111000
C	0.23561200	6.45490400	4.74773100
H	-0.94141900	6.25751700	4.25795700
H	0.42925000	6.29304200	5.79804500
O	-0.23835300	8.16713300	3.20328600
C	0.76584400	5.41688000	3.75340400
H	1.00435400	4.48593600	4.26089300
O	-1.34366000	5.98137500	2.89992600
H	1.66703900	5.75559300	3.24187300
H	0.26047000	8.61598600	5.08365400
C	-0.32727600	5.16339800	2.70064200
O	-0.21511100	4.34600800	1.82841100

A = **O;** XH = **SH**

REAGENT _C3=O_SH_VTZ

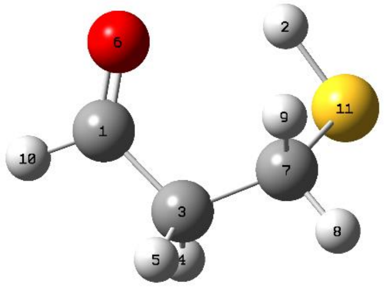

C	0.21952000	7.93714500	4.53504800
H	-0.66730600	6.64326500	2.04685500
C	0.28660700	6.48285200	4.91133100
H	-0.65667200	6.21405900	5.39359100
H	1.05767600	6.39965000	5.68666200
O	0.48025400	8.36519600	3.44359200
C	0.59242500	5.56515500	3.74294300
H	0.77534800	4.55344400	4.09759500
H	1.47924500	5.90222400	3.21194600
H	-0.07899600	8.62526700	5.34737000
S	-0.77888100	5.40962300	2.55578000

TSI-CHAT _C3=O_SH_VTZ

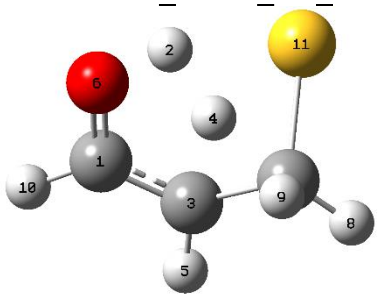

C	0.21148700	7.85471500	4.50354500
H	-0.35908400	7.41367700	2.76737400
C	0.37441100	6.50363100	4.82227800
H	-0.87419400	6.25291000	4.47373100
H	0.63828500	6.28591400	5.84931800
O	-0.05791400	8.25996600	3.31553800
C	0.71391700	5.52812300	3.71438100
H	0.89503300	4.53558100	4.11436300
H	1.57137600	5.83980900	3.11847800
H	0.18079100	8.62212300	5.27267000
S	-0.86376400	5.56667600	2.75821500

PRODUCT _C3=O_SH_VTZ

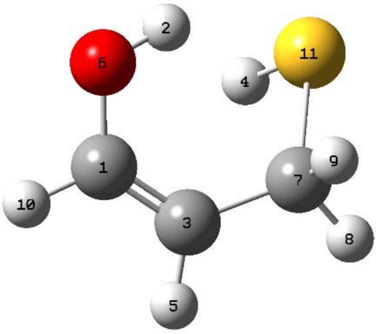

C	0.29553500	7.91093300	4.57722100
H	-0.30814300	7.72364100	2.77839800
C	0.60679000	6.64017600	4.82306600
H	-1.72987400	5.47519100	3.71973600
H	0.86320400	6.38485300	5.84101500
O	-0.06938800	8.43673900	3.38969700
C	0.66261000	5.54718300	3.80157800
H	0.84258500	4.58707000	4.27520000
H	1.46779900	5.71201300	3.08311000
H	0.31673900	8.65758900	5.35973100
S	-0.82648800	5.39277000	2.73275500

A = **CH$_2$; ** XH = **OOH**

REAGENT _C3=CH2_HOO_VTZ

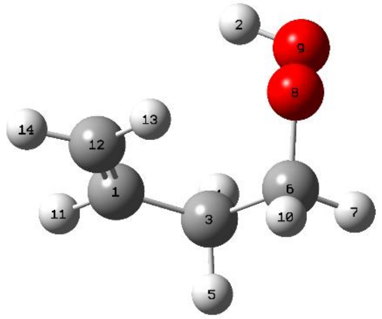

C	-0.03571200	7.87538800	4.47232600
H	-1.76883500	6.05565300	2.61995800
C	0.14776800	6.40917700	4.73311600
H	-0.81145500	5.97032300	5.01703200
H	0.79121900	6.28267000	5.60888800
C	0.75281900	5.59595400	3.60424900
H	0.83443800	4.54816900	3.90267400
O	0.04652700	5.67984500	2.37801200
O	-1.27921300	5.22292200	2.59996700
H	1.74611700	5.96476100	3.34129200
H	-0.47771800	8.43120700	5.29368500
C	0.28955200	8.53317400	3.36831500
H	0.72001600	8.04021500	2.50736900
H	0.12551700	9.59975100	3.29561900

PRODUCT _C3=CH2_HOO_VTZ

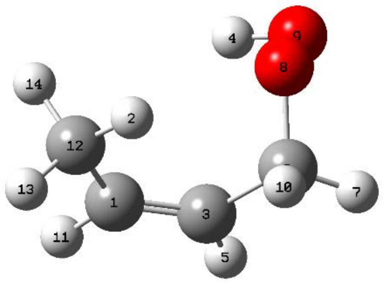

C	0.23670200	7.77578800	4.49804600
H	0.38142900	7.89548600	2.34827000
C	0.45943200	6.47296200	4.63600200
H	-1.93697300	5.70968400	3.82198200
H	0.49946200	6.05102500	5.63254500
C	0.63461800	5.52040100	3.48140600
H	0.86529800	4.51560300	3.83638000
O	-0.50437400	5.46377200	2.63378800
O	-1.57438700	4.92291800	3.39223200
H	1.43100500	5.84528200	2.80914800
H	0.10441900	8.36680900	5.39862500
C	0.15083800	8.52352300	3.20498400
H	0.83016500	9.37704600	3.21585300
H	-0.85480500	8.92342400	3.06086600

TSI-CHAT _C3=CH2_HOO_VTZ

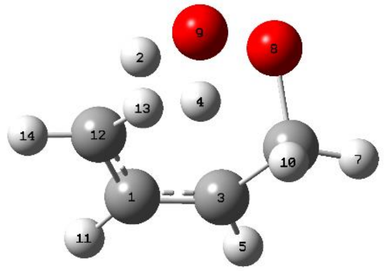

C	0.13385900	7.80622000	4.49207000
H	-1.06563600	7.18304800	2.92889100
C	0.36038600	6.45626400	4.78109600
H	-1.04586000	6.19697700	4.12842200
H	0.43879400	6.12210400	5.80327300
C	0.85004300	5.56549500	3.69314600
H	1.09326700	4.56961700	4.05497100
O	-0.20265900	5.39209800	2.64863700
O	-1.37247900	6.08316800	3.06520700
H	1.69334700	5.97788300	3.13431100
H	-0.14005900	8.44665600	5.32533400
C	-0.01041500	8.31347500	3.20516300
H	0.53506300	7.84438400	2.38756700
H	-0.25266600	9.35607100	3.06923200

A = **S;** XH = **OOH**

REAGENT_C3=S_HOO_VTZ

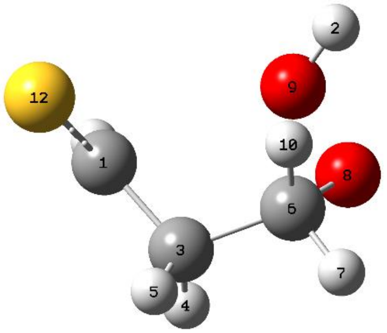

C	0.15176200	7.83991300	4.55974300
H	-1.21109100	6.29549100	1.75548200
C	0.46024700	6.42088100	4.92865400
H	-0.28216600	6.03242700	5.62701700
H	1.44524300	6.38861600	5.39477700
C	0.50917500	5.49141900	3.71281700
H	0.91107300	4.52177500	4.00710400
O	-0.76346700	5.17221300	3.19334900
O	-1.34615700	6.37335500	2.70843800
H	1.14369700	5.92511200	2.93465400
H	-0.89683400	8.12306800	4.59058300
S	1.25772900	8.91890100	4.10160600

PRODUCT _C3=S_HOO_VTZ

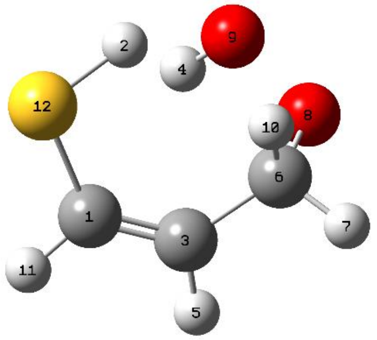

C	0.33890000	7.85410100	4.64546100
H	-0.04351600	7.78797000	2.33012800
C	0.51857700	6.54418400	4.80268500
H	-1.86234200	6.19179000	3.52068300
H	0.54943600	6.16972800	5.81881500
C	0.67734000	5.53592900	3.70836500
H	1.33680900	4.72858100	4.02676700
O	-0.53591200	4.86206600	3.39339800
O	-1.39395100	5.80347900	2.76864800
H	1.08676600	5.98130500	2.79966300
H	0.26801700	8.48820000	5.52047000
S	0.19622500	8.80067900	3.17384900

TSI-CHAT _C3=S_HOO_VTZ

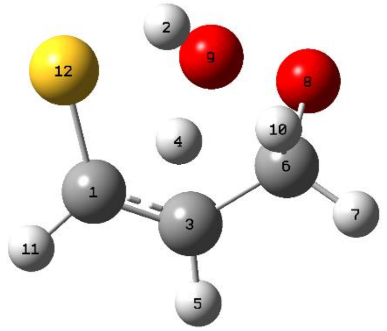

C	0.26271900	7.82093900	4.52546600
H	-0.93106300	6.90913100	2.54823100
C	0.28829500	6.44826500	4.77247700
H	-1.04315500	6.36510500	4.06347400
H	0.31195000	6.11030900	5.79844200
C	0.76207000	5.50632200	3.70835400
H	1.15658100	4.58071000	4.11785900
O	-0.36318500	5.06651600	2.86101600
O	-1.35822400	6.07178000	2.98147200
H	1.48205600	5.98683400	3.04582400
H	0.22481900	8.47415800	5.39375700
S	0.16141700	8.54824600	3.01071500

A = NH; XH = OOH

REAGENT _C3=HN_HOO__VTZ

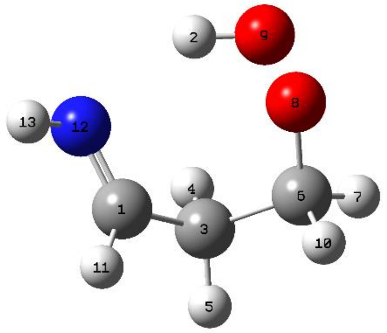

C	0.18928700	7.85409500	4.27370400
H	-1.33801600	6.59648900	2.54828600
C	0.07651700	6.44999100	4.78472700
H	-0.97680800	6.21108300	4.91643200
H	0.58158500	6.34126700	5.74426100
C	0.69221000	5.45771800	3.78930700
H	0.42092000	4.43926300	4.07677400
O	0.30635300	5.71187700	2.45797700
O	-1.11131200	5.66048500	2.39109300
H	1.77968300	5.54324400	3.76238200
H	1.07650800	8.42641900	4.55906200
N	-0.69592600	8.32096200	3.50061900
H	-0.47750800	9.26695800	3.19695000

PRODUCT _C3=HN_HOO_VTZ

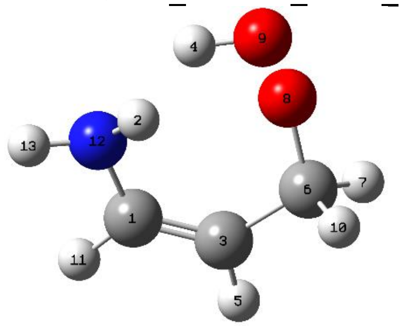

C	0.16094300	7.78718800	4.44970100
H	-0.17790000	7.74383200	2.43622200
C	0.68551600	6.58955300	4.69887800
H	-1.67377000	6.41345800	3.26440700
H	1.10972600	6.42996700	5.68057100
C	0.72251200	5.41659200	3.76628500
H	0.47230700	4.49735100	4.30214800
O	-0.13130900	5.54182300	2.64197400
O	-1.47262600	5.47558000	3.10225800
H	1.71252300	5.28740300	3.31961400
H	0.16151600	8.53321200	5.23381800
N	-0.50007900	8.18903200	3.28330500
H	-0.59643600	9.18512600	3.18141600

TSI-CHAT _C3=HN_HOO__VTZ

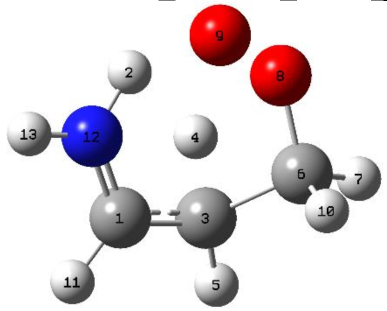

C	0.20571000	7.82740300	4.35820300
H	-0.78718300	7.47972900	2.66720300
C	0.18391500	6.45856400	4.72839000
H	-0.92323400	6.14589100	4.29038700
H	0.45065700	6.25793800	5.75657300
C	0.75126600	5.44375000	3.69828300
H	0.63519900	4.44987100	4.14113000
O	0.00443700	5.59200900	2.52773600
O	-1.35782000	5.78689200	2.91836300
H	1.79521700	5.61580800	3.43297100
H	0.54752200	8.60902000	5.02918500
N	-0.27885500	8.20731200	3.20796800
H	-0.32485100	9.18028600	2.95755200

A = **NH;** XH = **SH**

REAGENT _C3=NH_SH_VTZ

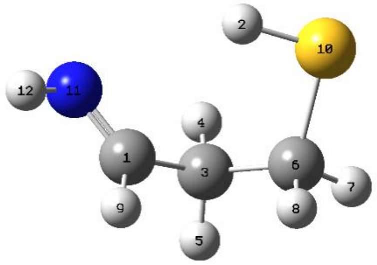

C	0.43427900	1.50378400	0.74553000
H	-0.33973300	0.26811700	-1.49637700
C	0.25347200	0.08978100	1.20154900
H	-0.80774700	-0.11092100	1.33719000
H	0.76418800	-0.03268900	2.16087100
C	0.86601000	-0.89098600	0.20220000
H	0.87953100	-1.89481700	0.62068400
H	1.89485000	-0.61429100	-0.02590200
H	1.47481400	1.84239600	0.66877700
S	-0.08618000	-1.03966400	-1.34380400
N	-0.54579000	2.24137100	0.44360400
H	-0.23219700	3.16308100	0.14280100

TSI-CHAT _C3=NH_SH_VTZ

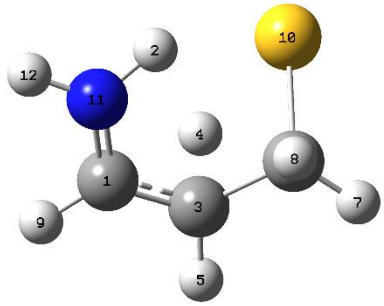

C	0.24201500	7.86143100	4.52056200
H	-0.14869500	7.58982500	2.57859100
C	0.32923500	6.49380900	4.82208800
H	-0.90164700	6.22683200	4.52034600
H	0.59704600	6.25986400	5.84507500
C	0.67710500	5.51780000	3.71644500
H	0.97396600	4.55873200	4.13104400
H	1.47703500	5.88032200	3.06839500
H	0.20081400	8.60128600	5.31100600
S	-0.95027100	5.39353700	2.85735300
N	0.07418400	8.29649500	3.28900700
H	-0.16337500	9.25729400	3.11160500

PRODUCT_C3=NH_SH_VTZ

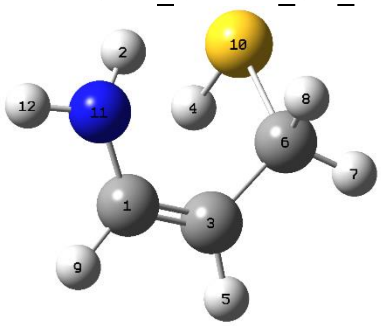

C	0.52420700	1.46769600	0.91354900
H	0.00245900	1.34914100	-1.04456700
C	0.68630600	0.17472000	1.20622200
H	-1.60589900	-1.15709000	0.27092100
H	0.77692600	-0.11292300	2.24285600
C	0.77456400	-0.89107900	0.16640200
H	1.08924100	-1.83679000	0.59618500
H	1.49266600	-0.62214100	-0.61111600
H	0.51613300	2.20613400	1.70486400
S	-0.78131200	-1.18364800	-0.78427700
N	0.41092600	1.97676800	-0.36637500
H	0.05108300	2.91267600	-0.43642000

<u>Optimized Structures of Scheme S4</u>

Scheme 4. Double *I-CHAT* tautomerization of the 2,6-diketo-4-hydroxymethyl heptane (4-hydroxymethyl -2,6-heptane-dione) - a KHP-model of the n-heptane combustion. The *I-CHAT*-catalyst (OH of the encircled CH_2OH group) relay-transfers its H atom to the first carbonyl group to catalyze its tautomerization and recovers itself by receiving H-atom from the α-carbon backbone; then the same process occurs with the second carbonyl oxygen, again recovering the hydroxymethyl catalyst group.

- Geometries and Energies from M06-2X/cc-pVTZ calculations.

Di-Keto

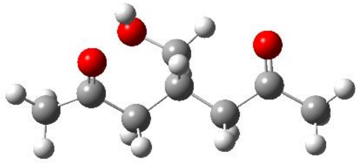

C	3.02810300	5.49182600	7.44972400
H	4.65199900	5.77523600	5.36935400
C	1.90597000	5.12760600	6.49226300
H	1.31809200	6.01913800	6.25925400
H	1.24867200	4.45216500	7.05031000
O	4.01079400	4.79921000	7.55978300
C	2.40275800	4.45487800	5.21650400
O	3.96737100	6.32914200	4.98051300
H	3.15926200	3.71668900	5.49048900
C	1.27510800	3.73897100	4.48653100
H	0.71727900	3.09890600	5.17838200
H	0.54067400	4.44992100	4.09270400
C	2.83235300	6.73893800	8.26648300
H	1.83830200	6.74973600	8.71538500
H	3.59839900	6.81595900	9.03201900
H	2.89501700	7.59399900	7.59033300
C	1.72819900	2.83372700	3.35544100
O	2.88681400	2.54902400	3.18803000
C	0.63799000	2.29729400	2.46147600
H	0.24507800	3.11407800	1.85301200
H	1.03556200	1.52233600	1.81322300
H	-0.19111600	1.91028400	3.05425800
C	3.04441800	5.49753400	4.30182700
H	3.51270900	5.00450800	3.44787700
H	2.26907300	6.17066000	3.92592900

Sum of electronic and zero-point Energies=	-538.718595
Sum of electronic and thermal Energies=	-538.705388
Sum of electronic and thermal Enthalpies=	-538.704444
Sum of electronic and thermal Free Energies=	-538.759231

TS1

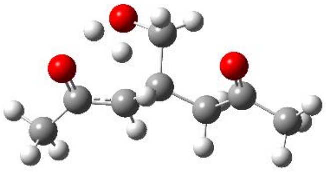

υ1= $-1377.73 cm^{-1}$

C	3.02011900	5.74691300	7.25080800
H	4.35462500	6.09544300	5.66944300
C	2.09810400	5.50987400	6.21064300
H	2.89125200	6.53687700	5.42506600
H	1.04718200	5.63311300	6.43288200
O	4.26790500	5.64699000	7.05343300
C	2.48584400	4.50957900	5.12283100
O	3.86958500	6.43508200	4.78510800
H	3.04568900	3.68622200	5.57120700
C	1.25548700	3.94409500	4.43266500
H	0.58509200	3.47466500	5.15740700
H	0.67186200	4.74982500	3.97149700
C	2.56050100	6.21880300	8.60188500
H	2.68856300	5.39563800	9.30668200
H	3.18781200	7.04115200	8.93989500
H	1.51436600	6.51450100	8.59787400
C	1.56792400	2.93253000	3.35151100
O	2.70068000	2.71001700	3.00018400
C	0.38920200	2.21603300	2.74267200
H	-0.38249800	2.92826800	2.44974200
H	0.71051000	1.63187500	1.88577200
H	-0.05139400	1.55438400	3.49055200
C	3.43718300	5.22420200	4.12592600
H	4.30315300	4.62508300	3.87183200
H	2.92686400	5.53138700	3.21363000

Sum of electronic and zero-point Energies=	-538.657216
Sum of electronic and thermal Energies=	-538.645259
Sum of electronic and thermal Enthalpies=	-538.644315
Sum of electronic and thermal Free Energies=	-538.696065

Keto-Enol

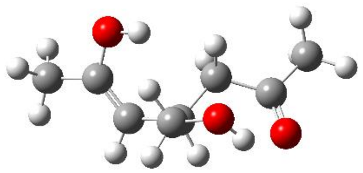

C	2.72072000	5.27791400	7.20162700
H	2.03171400	5.77887800	5.47541300
C	3.93227300	4.90968900	6.78521100
H	5.65420300	5.72017200	3.32067900
H	4.62844200	4.59805900	7.55067900
O	1.71946300	5.71501900	6.38173000
C	4.42660900	4.92340500	5.35885300
O	5.05809800	6.44714400	3.53594100
H	5.37819700	4.38966400	5.33980400
C	3.51082800	4.20045800	4.35944500
H	2.85530400	3.48575400	4.86212300
H	2.85557400	4.90135200	3.82562700
C	2.26291600	5.25914100	8.62074100
H	1.39131700	4.61142400	8.72123400
H	3.05120200	4.90370700	9.27799200
H	1.96195800	6.26133800	8.92713800
C	4.25442300	3.46624800	3.25897600
O	5.43568400	3.62170700	3.06085600
C	3.43077500	2.52256000	2.42315500
H	4.00209000	2.18890500	1.56249100
H	3.15216400	1.66140400	3.03325300
H	2.50599900	3.00364200	2.10440400
C	4.71716400	6.35941500	4.90146100
H	5.50170000	6.78056100	5.53728000
H	3.82870700	6.98265900	5.03669600

Sum of electronic and zero-point Energies= -538.701535
Sum of electronic and thermal Energies= -538.688526
Sum of electronic and thermal Enthalpies= -538.687582
Sum of electronic and thermal Free Energies= -538.741121

TS2

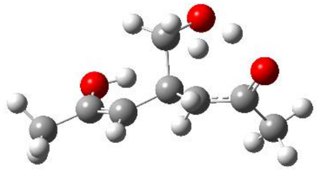

υ1 -1393.64 cm-1

C	2.37639000	-0.31873600	-0.02570100
H	1.11215400	-0.25318200	1.42401700
C	1.50504200	-0.03166300	-0.99389000
H	-1.97913400	1.46168300	0.14381900
H	1.86168800	-0.08694300	-2.01174500
O	2.06758800	-0.30758300	1.30316700
C	0.07912500	0.38653700	-0.75927400
O	-1.16118500	1.75539700	0.76817300
H	-0.42670100	0.45711800	-1.72286700
C	-0.73697900	-0.57697400	0.12309100
H	-0.26809400	-1.51095600	0.40348300
H	-1.02271900	0.61739200	1.02379400
C	3.80646900	-0.68155700	-0.23914900
H	4.00401000	-1.67505900	0.16434900
H	4.05747100	-0.66493500	-1.29593600
H	4.44889700	0.02055000	0.29355000
C	-2.13494400	-0.61281800	-0.09917800
O	-2.76207900	0.42097700	-0.47198600
C	-2.92883200	-1.85896700	0.16926200
H	-3.86468700	-1.60456300	0.66184900
H	-3.17207100	-2.31510000	-0.79200900
H	-2.36788800	-2.57833900	0.76044500
C	-0.00890800	1.79289300	-0.10039000
H	-0.13462300	2.58929600	-0.82765200
H	0.85292700	2.00042300	0.53144900

Sum of electronic and zero-point Energies= -538.640745
Sum of electronic and thermal Energies= -538.629139
Sum of electronic and thermal Enthalpies= -538.628195
Sum of electronic and thermal Free Energies= -538.678257

Di-Enol

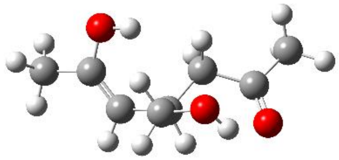

C	2.71374700	5.24382600	7.18530900
H	2.04060700	5.51957000	5.40159300
C	3.94834600	4.88725200	6.82995900
H	5.67156700	4.55721500	3.17905600
H	4.62392200	4.60814400	7.62503500
O	1.73708400	5.62417700	6.30928600
C	4.47364600	4.88463100	5.41350400
O	5.18796900	6.41209100	3.62342900
H	5.48201900	4.45876100	5.43270000
C	3.63941600	4.02915500	4.48099900
H	2.67301200	3.68375500	4.82442300
H	4.47060800	6.31093800	2.99106400
C	2.20540500	5.27932700	8.58669800
H	1.35498200	4.60479300	8.69091400
H	2.98205300	4.98946000	9.28860100
H	1.85754300	6.28384900	8.82956900
C	4.07524100	3.50617200	3.32837100
O	5.29052400	3.75917800	2.78254200
C	3.30661200	2.52717300	2.50468400
H	3.15535300	2.91859800	1.49852700
H	3.88099900	1.60477500	2.41054400
H	2.34395400	2.30416800	2.95585300
C	4.64697000	6.33624300	4.93715100
H	5.35299300	6.84320400	5.59316200
H	3.69972900	6.87985200	4.99003000

Sum of electronic and zero-point Energies= -538.689376
Sum of electronic and thermal Energies= -538.676736
Sum of electronic and thermal Enthalpies= -538.675792
Sum of electronic and thermal Free Energies= -538.727696

Optimized Structures of Figure S1

Figure 1. An illustrative example of the more complex long-range and sequential keto-enol tautomerization of a model 2,4-diketo-6-hydroxy hexane - an intermediate of the n-heptane oxidation. The *I-CHAT* catalyst (OH, or rather encircled CH_2OH - group), transfers one of its H-atom to the distant carbonyl oxygen and concomitantly accepts an H-atom from the α-methylene group to produce 2- enol-4-keto-6-hydroxy hexane product. Another *I-CHAT* rearrangement of regular type provided in Scheme 2, can occur with the second carbonyl group, again, regenerating the catalyst moiety and forming 2-keto-4-enol-6-hydroxy hexane isomer.

■ Geometries and Energies from M06-2X/cc-pVTZ calculations.

Di-Keto-OH

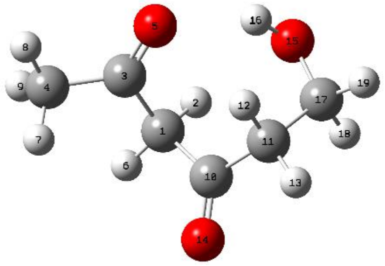

C	0.87934700	8.46018300	5.53986000
H	1.90896100	8.32975900	5.20137100
C	-0.02512000	7.85000200	4.48410600
C	-1.12725600	6.96030400	4.98069500
O	0.12657100	8.08723800	3.30727400
H	0.72733100	7.99750300	6.51080900
H	-1.72235300	7.50150100	5.71875100
H	-1.74895000	6.62829300	4.15523800
H	-0.69241100	6.10167600	5.49516900
C	0.63558400	9.96064000	5.69972400
C	0.81404500	10.85250400	4.49348100
H	0.09650500	10.55484200	3.72508800
H	0.57629100	11.86639200	4.81052100
O	0.31429100	10.40128200	6.77364200
O	2.59059100	9.57699800	3.34335400
H	1.81390900	9.14935000	2.95812400
C	2.21863400	10.82544000	3.88204100
H	2.96050700	11.07551700	4.64198100
H	2.26123400	11.60350700	3.11446300

Sum of electronic and zero-point Energies= -460.154895
Sum of electronic and thermal Energies= -460.144888
Sum of electronic and thermal Enthalpies= -460.143944
Sum of electronic and thermal Free Energies= -460.191087

TS1

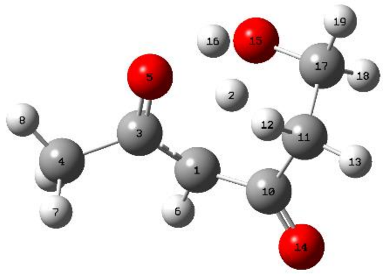

υ1=-1264.9595 cm-1

C	0.64713000	8.65062400	5.58705900
H	1.87375900	8.78852400	4.68812300
C	-0.08400500	8.04233900	4.50548100
C	-0.94595400	6.85171300	4.81768000
O	0.00369200	8.41088400	3.31073700
H	0.60326900	8.11096300	6.52416900
H	-1.85920800	7.19996600	5.30301500
H	-1.20001500	6.32055700	3.90522700
H	-0.44099200	6.19002500	5.51977400
C	0.70478700	10.10673400	5.80809300
C	0.83663600	10.99674000	4.58311500
H	0.01823500	10.82061400	3.88513000
H	0.82798700	12.03405400	4.90736500
O	0.76553000	10.58270500	6.92321000
O	2.26061100	9.24173900	3.68240400
H	1.35327500	8.95560100	3.23018000
C	2.16651500	10.66938700	3.90462500
H	3.00897900	10.93766800	4.53863800
H	2.26936900	11.16111300	2.94078400

Sum of electronic and zero-point Energies= -460.108326
Sum of electronic and thermal Energies= -460.099662
Sum of electronic and thermal Enthalpies= -460.098718
Sum of electronic and thermal Free Energies= -460.142073

Enol-Keto-OH

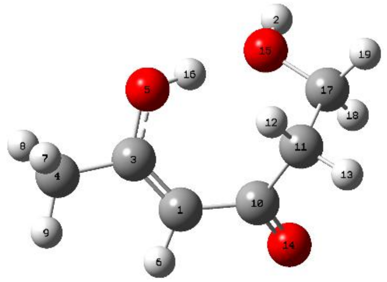

C	0.11785800	8.67477100	5.81673900
H	3.29492600	9.41699200	3.03405200
C	-0.27381300	7.92808100	4.76519300
C	-1.17416700	6.74626600	4.91000800
O	0.08056400	8.11031400	3.48481000
H	-0.14390600	8.31643800	6.80219700
H	-2.04117300	6.88077400	4.26198300
H	-0.65774000	5.84606800	4.57631000
H	-1.50619100	6.62112800	5.93585100
C	0.89146000	9.91653800	5.82058900
C	0.89088600	10.85140500	4.61245900
H	0.10843700	10.60630500	3.89481200
H	0.70283500	11.84965100	5.00644800
O	1.49521800	10.27411300	6.81243700
O	2.39490200	9.55767500	3.33287500
H	0.89540500	8.63841200	3.39746900
C	2.24444500	10.84533400	3.93395700
H	3.02485800	11.01241500	4.67819500
H	2.29997600	11.62212400	3.16926400

Sum of electronic and zero-point Energies= -460.150194
Sum of electronic and thermal Energies= -460.140261
Sum of electronic and thermal Enthalpies= -460.139317
Sum of electronic and thermal Free Energies= -460.185288

TS2

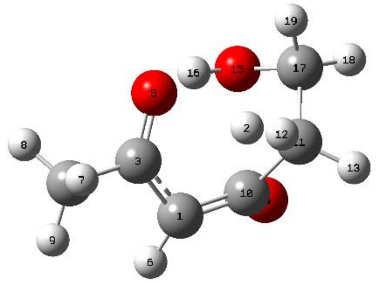

υ1=-976.8849

C	0.33848000	8.61808000	5.82033600
H	2.80571100	9.53139800	4.93380400
C	-0.16848100	8.04024300	4.61715300
C	-1.18306100	6.93272500	4.69239900
O	0.27869900	8.35996400	3.49003500
H	0.43364800	7.98229500	6.68997600
H	-2.14222600	7.33835900	4.36498900
H	-0.91311400	6.12921600	4.01058900
H	-1.29836100	6.55516800	5.70518100
C	1.16515200	9.71872400	5.68469400
C	0.84742700	10.81107100	4.67869800
H	-0.13776700	10.69603400	4.24170600
H	0.90105100	11.75508000	5.22121000
O	2.35287100	9.81926500	6.16700600
O	2.66541300	9.48268600	3.78606500
H	1.91933100	8.81746700	3.60942200
C	1.95257100	10.74889800	3.61032000
H	2.70410400	11.52408700	3.71340300
H	1.54467900	10.74389100	2.60517500

Sum of electronic and zero-point Energies= -460.096204
Sum of electronic and thermal Energies= -460.087509
Sum of electronic and thermal Enthalpies= -460.086564
Sum of electronic and thermal Free Energies= -460.129812

Keto- Enol-OH

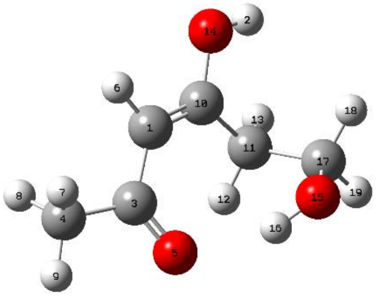

C	0.31577700	8.66096400	5.61691600
H	1.77919200	11.27271000	6.46909100
C	-0.23426300	7.90845700	4.49226600
C	-1.16677800	6.78163200	4.85988700
O	0.02920100	8.13153100	3.31956000
H	0.21756100	8.23112500	6.60470000
H	-0.63144600	6.05645000	5.47501900
H	-1.99702500	7.15964100	5.45714100
H	-1.53696700	6.29950000	3.96069200
C	0.90786900	9.86318900	5.51775100
C	1.07777700	10.67759300	4.27482700
H	0.30629800	10.41393200	3.55607500
H	0.95663600	11.73479600	4.53039200
O	1.39759400	10.40911000	6.65089600
O	2.69578900	9.14260900	3.27568500
H	1.85152200	8.72452700	3.05162900
C	2.45405300	10.47737800	3.62772800
H	3.24539400	10.77807000	4.31953700
H	2.51343900	11.13655100	2.75530500

Sum of electronic and zero-point Energies=	-460.148388
Sum of electronic and thermal Energies=	-460.138381
Sum of electronic and thermal Enthalpies=	-460.137436
Sum of electronic and thermal Free Energies=	-460.183771

Optimized Structures from Figure S2

Figure 2. Potential energy diagram for I-CHAT, Direct, Korcek, and OH dissociation KHP pathways. Enthalpies (kcal/mol) are relative to the KHP baseline.

KHP

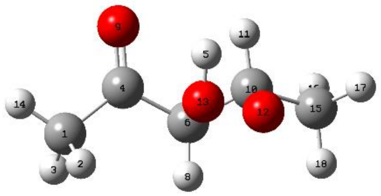

C	-1.05648882	8.66971203	4.52389687
H	-0.15012313	9.10313250	4.09839623
H	-1.01913605	8.83230890	5.60242859
C	-1.06319370	7.19579558	4.22979330
H	0.44838892	6.70159003	1.65020128
C	0.05443434	6.36992089	4.84500792
H	-0.38785513	5.83460407	5.68932767
H	0.85289248	7.00545711	5.23006843
O	-1.92262315	6.67071615	3.56520163
C	0.63968167	5.35591656	3.87327094
H	-0.17517721	4.92199361	3.28480412
O	1.56825070	5.98298673	2.98934863
O	0.94094528	7.09942710	2.38152577
H	-1.93668631	9.14488821	4.10163659
C	1.44547616	4.27176612	4.55807421
H	0.79224957	3.66811576	5.18578360
H	1.91648799	3.62457784	3.82061001
H	2.22306740	4.71416179	5.18045821

Sum of electronic and zero-point Energies=	-421.969248
Sum of electronic and thermal Energies=	-421.959563
Sum of electronic and thermal Enthalpies=	-421.958619
Sum of electronic and thermal Free Energies=	-422.003828

$TS_{korcek-1}$

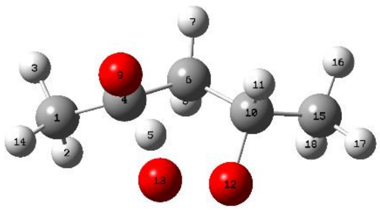

C	-0.72883200	8.95827768	4.27009037
H	0.27984743	9.22886917	4.57265786
H	-1.40384830	9.11132741	5.11451636
C	-0.82074838	7.51523193	3.87536826
H	-0.88082540	6.94583698	2.31426777
C	-0.14787789	6.48165520	4.75281711
H	-0.89667763	6.04247386	5.41049780
H	0.65385637	6.92254820	5.34549896
O	-1.83818214	7.12720757	3.15395362
C	0.41924365	5.44229416	3.79408062
H	-0.40634564	4.89986543	3.32431949
O	1.09943839	6.20089836	2.78909919
O	0.25070606	7.28102286	2.48437127
H	-1.06221125	9.58405619	3.44649625
C	1.42837013	4.49127557	4.38804146
H	0.94947237	3.87249021	5.14545310
H	1.83687714	3.83961133	3.61780729
H	2.24507409	5.04557890	4.84870919

Sum of electronic and zero-point Energies=	-421.916914
Sum of electronic and thermal Energies=	-421.908787
Sum of electronic and thermal Enthalpies=	-421.907843
Sum of electronic and thermal Free Energies=	-421.948953

Cyclic Korcek

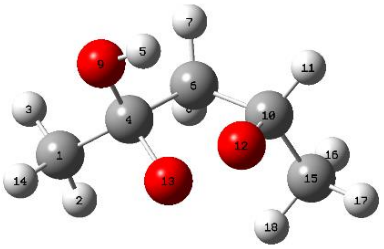

C	-0.25166861	2.16667953	0.82867004
H	0.63066809	2.11725875	1.46277673
H	-1.12905408	2.36350045	1.44143261
C	-0.44224112	0.86196945	0.09716536
H	-1.54718890	0.25706370	-1.35852228
C	-0.51546042	-0.40040649	0.97658136
H	-1.54808019	-0.72311329	1.07858925
H	-0.10714949	-0.20850405	1.96734604
O	-1.56405461	0.98857479	-0.73117332
C	0.36438944	-1.39425658	0.20454084
H	-0.16590409	-2.31046591	-0.05345725
O	0.58440340	-0.73927128	-1.05377760
O	0.72110820	0.63461942	-0.68448213
H	-0.14012983	2.97149385	0.10549604
C	1.68131517	-1.69779732	0.88884752
H	1.50488168	-2.19535854	1.84266595
H	2.29758385	-2.34205154	0.26466297
H	2.22047953	-0.76894591	1.07511186

Sum of electronic and zero-point Energies=	-421.978741
Sum of electronic and thermal Energies=	-421.970571
Sum of electronic and thermal Enthalpies=	-421.969627
Sum of electronic and thermal Free Energies=	-422.011027

$TS_{korcek-2}$

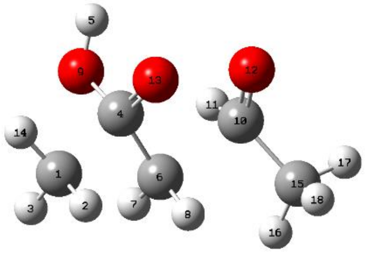

C	-0.78071325	8.79143895	4.65246181
H	0.13409653	9.14140155	5.11215315
H	-1.53057590	8.48550047	5.37148097
C	-0.43282643	7.59359546	3.51267980
H	-1.47551905	7.34065017	1.95725745
C	-0.09794325	6.63903642	4.59748774
H	-0.90917154	6.22033753	5.18422466
H	0.79262890	6.89802708	5.15672733
O	-1.64622222	7.26101453	2.90292271
C	0.54743423	5.27152743	3.48024169
H	-0.44135696	4.81012826	3.31232343
O	1.08283514	5.85097405	2.50360848
O	0.52082410	7.97017615	2.78413074
H	-1.18851354	9.52904744	3.97103700
C	1.43986562	4.55775687	4.49684272
H	0.93023789	4.36069181	5.43930018
H	1.73270775	3.60136256	4.06285862
H	2.33832372	5.14648443	4.67169622

Sum of electronic and zero-point Energies=	-421.897358
Sum of electronic and thermal Energies=	-421.888102
Sum of electronic and thermal Enthalpies=	-421.887158
Sum of electronic and thermal Free Energies=	-421.930698

Propionic Acid

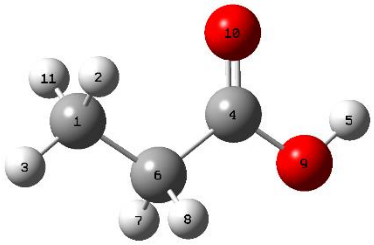

C	-0.58636688	9.13450843	5.14524161
H	0.15952548	9.75044235	4.64694427
H	-0.43072299	9.20006096	6.22025651
C	-0.69643551	7.55065787	3.19951796
H	-0.75074895	6.25042730	1.84676000
C	-0.48861161	7.69250931	4.68191120
H	-1.22562409	7.05480013	5.17328239
H	0.48362087	7.25415939	4.91419754
O	-0.60871496	6.26459064	2.80268101
O	-0.91517754	8.44464643	2.43058185
H	-1.56406481	9.54950217	4.90875466

Sum of electronic and zero-point Energies=	-268.306274
Sum of electronic and thermal Energies=	-268.300584
Sum of electronic and thermal Enthalpies=	-268.299640
Sum of electronic and thermal Free Energies=	-268.335374

Acetaldehyde

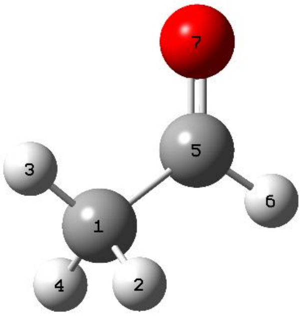

C	1.16811653	-0.14887784	0.00097315
H	1.69583894	0.20813033	0.88722455
H	1.15146845	-1.23495077	-0.01583290
H	1.70758561	0.23527777	-0.86665735
C	-0.22673878	0.39904353	0.00014625
H	-0.30674145	1.50306032	0.01542529
O	-1.21946130	-0.27484333	-0.01547498

Sum of electronic and zero-point Energies=	-153.764884
Sum of electronic and thermal Energies=	-153.760994
Sum of electronic and thermal Enthalpies=	-153.760050
Sum of electronic and thermal Free Energies=	-153.789815

TS$_{\text{I-CHAT}}$

(Keto-Hydroperoxide to Enol-Hydroperoxide)

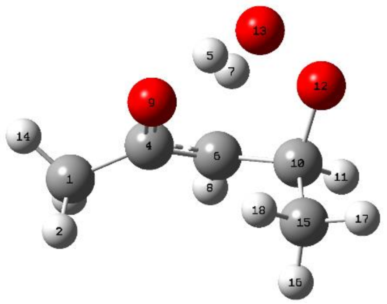

C	0.35956215	8.90046244	5.41337308
H	1.35019425	9.32890507	5.24745718
H	0.32183401	8.50820694	6.42617678
C	0.15269840	7.82408260	4.39478121
H	-0.86107500	7.16774816	2.79982183
C	0.24256783	6.45238692	4.72471505
H	-1.04015421	6.22806038	4.09239464
H	0.43174711	6.20232616	5.75766405
O	-0.19668461	8.18217660	3.22531309
C	0.77020772	5.46982701	3.69731163
H	0.85967761	4.49424609	4.17741502
O	-0.21759288	5.30914326	2.62152553
O	-1.33895668	6.12410996	2.92783483
H	-0.37308336	9.69263382	5.27769804
C	2.07732576	5.84490823	3.02661744
H	2.88339179	5.83683644	3.75991560
H	2.31427515	5.12701408	2.24188924
H	2.00378596	6.83593083	2.58162174

Sum of electronic and zero-point Energies=	-421.906572
Sum of electronic and thermal Energies=	-421.898254
Sum of electronic and thermal Enthalpies=	-421.897310
Sum of electronic and thermal Free Energies=	-421.939763

ENOL

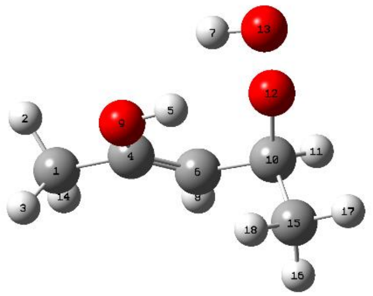

C	0.19968686	9.00493894	5.46597543
H	-0.76651503	9.50215938	5.37707714
H	0.96924140	9.75528015	5.28227683
C	0.31137908	7.91931857	4.44916526
H	0.16397634	7.67594498	2.55587374
C	0.51925898	6.63018237	4.73398709
H	-1.64582798	6.45595724	3.47707877
H	0.60514735	6.35204995	5.77426754
O	0.16360526	8.40926237	3.18940706
C	0.68177686	5.54232852	3.70593947
H	0.37919852	4.58270886	4.12902011
O	-0.14261940	5.78006098	2.55555361
O	-1.50271878	5.66489091	2.93412776
H	0.31340532	8.61239224	6.47233006
C	2.09512818	5.45376425	3.15284072
H	2.78612969	5.23802930	3.96548904
H	2.16610664	4.66692990	2.40379902
H	2.38543070	6.40453809	2.70546733

Sum of electronic and zero-point Energies=	-421.957108
Sum of electronic and thermal Energies=	-421.947724
Sum of electronic and thermal Enthalpies=	-421.946779
Sum of electronic and thermal Free Energies=	-421.99108

TS$_{\text{Direct}}$

(Same as TS$_{\text{dir}}$ from Scheme S2)

TS$_{enol}$

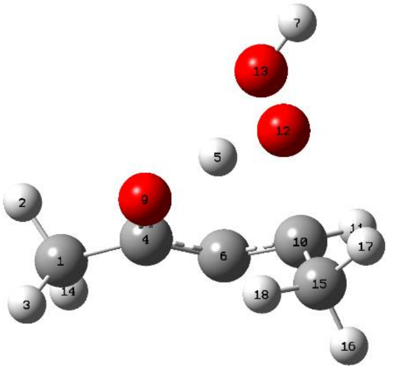

C	0.32012838	9.01448944	5.46895438
H	-0.58276086	9.57798838	5.23696201
H	1.16672773	9.69139816	5.34923941
C	0.47215529	7.89033314	4.49190146
H	0.02538781	7.09568736	2.65735593
C	0.53456877	6.55408751	4.89950797
H	-2.15032910	5.65292348	2.26697815
H	0.11564842	6.27578894	5.85461859
O	0.45070880	8.18322125	3.25233375
C	0.80954597	5.57501580	3.93558843
H	0.44649622	4.57341423	4.14577535
O	-0.34239169	6.04447015	2.45937196
O	-1.67171455	6.13732633	2.94856941
H	0.28777397	8.65883984	6.49523474
C	2.03413011	5.60716350	3.06897554
H	2.82983405	5.11081196	3.62802580
H	1.88210289	5.05825903	2.14161906
H	2.35034079	6.62379749	2.85315004

Sum of electronic and zero-point Energies=	-421.908241
Sum of electronic and thermal Energies=	-421.898730
Sum of electronic and thermal Enthalpies=	-421.897786
Sum of electronic and thermal Free Energies=	-421.942391

cis-Pentenone

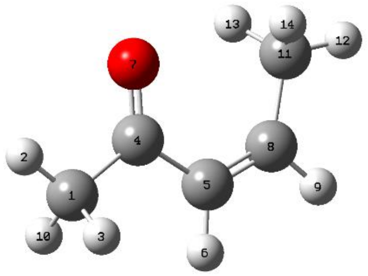

C	-0.05102305	8.95351707	5.31895152
H	-0.21318045	9.87239486	4.76431235
H	0.59033332	9.14436062	6.18093657
C	0.58696641	7.91723820	4.42630164
C	0.86977809	6.60688299	5.05802939
H	0.58882839	6.50053158	6.09883234
O	0.84704605	8.15332311	3.26644872
C	1.43921458	5.57697976	4.42869486
H	1.58885444	4.67362549	5.01093911
H	-0.99990750	8.57584583	5.70343309
C	1.89301921	5.53631496	3.00435651
H	2.33688985	4.57322289	2.76564123
H	1.05885995	5.72664807	2.32903062
H	2.61524571	6.32784355	2.80604605

Sum of electronic and zero-point Energies=	-270.410707
Sum of electronic and thermal Energies=	-270.403449
Sum of electronic and thermal Enthalpies=	-270.402505
Sum of electronic and thermal Free Energies=	-270.442268

H_2O_2

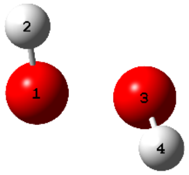

O	-2.71227191	6.91916792	-1.37454591
H	-2.93986057	7.69187478	-0.84518603
O	-1.29067077	6.98343818	-1.37460980
H	-1.06308655	6.21175923	-0.84375111

Sum of electronic and zero-point Energies=	-151.526501
Sum of electronic and thermal Energies=	-151.523291
Sum of electronic and thermal Enthalpies=	-151.522347
Sum of electronic and thermal Free Energies=	-151.548755

PN-2O4OJ

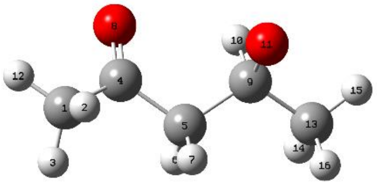

C	-1.09111647	8.66863541	4.58110312
H	-0.19922818	9.19903388	4.24151347
H	-1.10720192	8.73393231	5.67000387
C	-0.98848194	7.23218461	4.14668445
C	0.08673388	6.39246176	4.81552098
H	-0.37599350	5.91851741	5.68637925
H	0.89401367	7.02553857	5.18777522
O	-1.72751371	6.75234636	3.32518412
C	0.65479532	5.33114253	3.87507129
H	-0.19713187	4.86382716	3.34830345
O	1.36434271	5.90601314	2.85065750
H	-1.97822266	9.12976350	4.15794355
C	1.46014253	4.25077535	4.59044311
H	0.83777715	3.72525074	5.31353706
H	1.84803097	3.53673166	3.86752775
H	2.30031103	4.69990162	5.12045980

Sum of electronic and zero-point Energies=	-346.174047
Sum of electronic and thermal Energies=	-346.165552
Sum of electronic and thermal Enthalpies=	-346.164608
Sum of electronic and thermal Free Energies=	-346.208560

OH

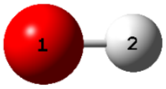

O	-2.65948180	6.94569331	-1.36253994
H	-2.98391769	7.86187196	-1.36253994

Sum of electronic and zero-point Energies=	-75.725224
Sum of electronic and thermal Energies=	-75.722863
Sum of electronic and thermal Enthalpies=	-75.721919
Sum of electronic and thermal Free Energies=	-75.742145

Optimized Structures from Table S3

Table 3. Rate constants for I-CHAT and Enol decomposition training reactions (Sec.3.4) employed to create a decision tree for three new reaction families.

KETO_24
(Same as KHP from Figure 2)

TS$_{I\text{-}CHAT}$
(KETO_24 to ENOL_24)

ENOL_24
(same as ENOL from Figure 2)

KETO_13

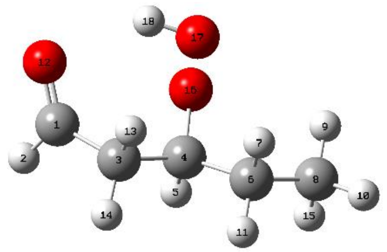

C	-1.96842019	0.22496461	-0.41260473
H	-2.52368282	-0.45960276	-1.07876675
C	-0.93722486	-0.38607049	0.49209900
C	0.41182250	-0.46943027	-0.24237309
H	0.35287359	-1.20766876	-1.04816574
C	1.54508190	-0.82951537	0.70562187
H	1.59844990	-0.06109350	1.47795597
C	2.88158135	-0.95276804	-0.01152187
H	3.14372189	-0.01102408	-0.49013837
H	3.67455300	-1.21517905	0.68681750
H	1.28555316	-1.76746446	1.20185510
O	-2.17328911	1.41006386	-0.46625437
H	-0.82683987	0.24219182	1.37495326
H	-1.23758746	-1.38900178	0.79495285
H	2.84328282	-1.72343424	-0.78281718
O	0.66924180	0.72580973	-0.96589667
O	0.68947900	1.81497353	-0.05248645
H	-0.22088460	2.14520827	-0.13250734

Sum of electronic and zero-point Energies=	-421.956788
Sum of electronic and thermal Energies=	-421.947493
Sum of electronic and thermal Enthalpies=	-421.946549
Sum of electronic and thermal Free Energies=	-421.991092

TS$_{I\text{-}CHAT}$
(KETO_13 to ENOL_13)

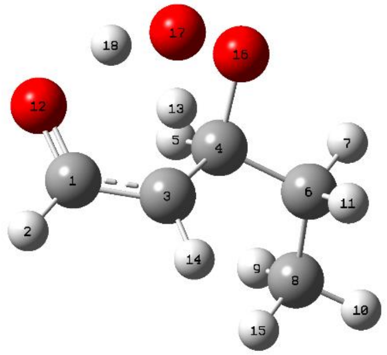

C	-1.38678127	-1.34194331	0.03522462
H	-1.73236533	-2.33371079	0.34437916
C	-0.24935248	-0.77219183	0.63225866
C	0.49861322	0.25295571	-0.18411427
H	0.41313945	0.01993690	-1.25022561
C	1.94789272	0.44487827	0.20739083
H	2.30682352	1.36022008	-0.26590770
C	2.81225314	-0.73552474	-0.21736817
H	2.76698130	-0.88076567	-1.29706508
H	3.85275376	-0.57019159	0.05577602
H	2.00020517	0.60000768	1.28645454
O	-2.10081353	-0.69873058	-0.78216351
H	-1.14431050	0.36033354	1.02075715
H	0.29520377	-1.34275170	1.36980295
H	2.48275893	-1.65949409	0.25725307
O	-0.14720965	1.56778337	-0.02161391
O	-1.47947167	1.30657789	0.39760680
H	-1.87319755	0.62290488	-0.36090255

Sum of electronic and zero-point Energies=	-421.895020
Sum of electronic and thermal Energies=	-421.887060
Sum of electronic and thermal Enthalpies=	-421.886116
Sum of electronic and thermal Free Energies=	-421.927553

ENOL_13

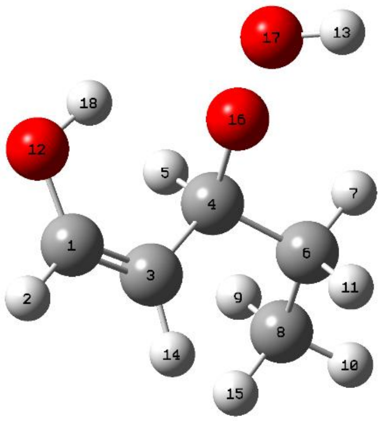

C	-1.63452748	-1.44078297	-0.07581279
H	-2.09667572	-2.40222453	0.10550913
C	-0.34216233	-1.22696436	0.15947718
C	0.34098150	0.07904095	-0.11984975
H	0.34467178	0.29190877	-1.19580374
C	1.76131299	0.13836743	0.41400896
H	2.11439014	1.16689246	0.33512930
C	2.70023102	-0.77950890	-0.35973408
H	2.72240099	-0.50660585	-1.41537695
H	3.71594862	-0.71045305	0.02556767
H	1.74441139	-0.12856743	1.47298956
O	-2.51460676	-0.53824376	-0.55251710
H	0.25479899	2.78228356	0.74991489
H	0.23994009	-2.03500140	0.57690493
H	2.38858117	-1.82151556	-0.29352050
O	-0.49491054	1.08463396	0.48714434
O	-0.10691141	2.35237961	-0.03386440
H	-2.12930442	0.34480907	-0.46383166

Sum of electronic and zero-point Energies= -421.945629
Sum of electronic and thermal Energies= -421.936141
Sum of electronic and thermal Enthalpies= -421.935196
Sum of electronic and thermal Free Energies= -421.980183

KETO_25

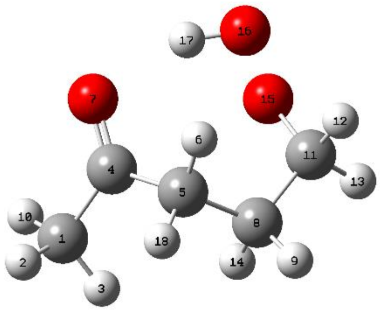

C	-1.25197835	8.44805958	4.83723777
H	-1.26183655	8.88008269	5.84050944
H	-1.75862722	7.48610218	4.89501963
C	0.18470129	8.29324966	4.41310234
C	0.95173337	7.13292697	5.00976946
H	2.01878874	7.32617151	4.90680872
O	0.69858966	9.09175061	3.66171118
C	0.58347729	5.78336192	4.37018121
H	0.82287213	4.98080671	5.06982228
H	-1.77221973	9.11542199	4.15725637
C	1.31143052	5.49198771	3.06597646
H	2.38130185	5.36097265	3.23146147
H	0.91523364	4.57588897	2.62393369
H	-0.49339385	5.73450174	4.19756997
O	1.13039269	6.49539828	2.08420003
O	2.23618567	7.38788536	2.14684023
H	1.83825978	8.16988926	2.57286415
H	0.70373407	7.09756422	6.07268362

Sum of electronic and zero-point Energies= -421.963025
Sum of electronic and thermal Energies= -421.953890
Sum of electronic and thermal Enthalpies= -421.952946
Sum of electronic and thermal Free Energies= -421.997793

TS$_{I\text{-}CHAT}$
(KETO_25 to ENOL_25)

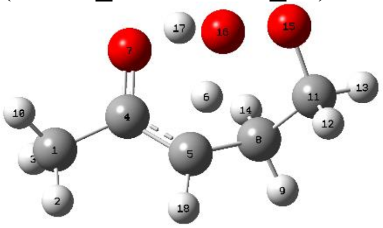

C	2.86285540	0.11271877	0.25668872
H	3.05651494	0.92992299	0.94624760
H	3.39487593	0.31331605	-0.67547929
C	1.40705402	-0.00829961	-0.06633006
C	0.46741869	0.96701721	0.31342842
H	-0.23387456	-0.08005666	0.97001843
O	1.01857320	-1.09933613	-0.60962813
C	-0.75273946	1.25387268	-0.57143816
H	-0.85306100	2.33252228	-0.68997650
H	3.23898393	-0.82728599	0.65493565
C	-2.05189339	0.70369198	-0.00680901
H	-2.23987158	1.09749252	0.99765341
H	-2.90516934	0.93152408	-0.64445343
H	-0.60073413	0.82044909	-1.56158461
O	-2.03674696	-0.71470955	0.03493896
O	-0.89742838	-1.12904530	0.79055955
H	-0.06223813	-1.30632749	-0.04780411
H	0.85804985	1.80499108	0.87411659

Sum of electronic and zero-point Energies=	-421.901110
Sum of electronic and thermal Energies=	-421.893212
Sum of electronic and thermal Enthalpies=	-421.892267
Sum of electronic and thermal Free Energies=	-421.933655

ENOL_25

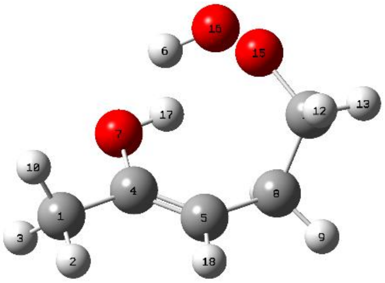

C	-0.82418837	8.94443239	5.24436347
H	-0.75890123	8.68379305	6.29679097
H	-1.85394272	9.20987936	5.00263520
C	-0.38134856	7.80775024	4.38751208
C	0.05349660	6.63090655	4.83315538
H	1.68986491	8.36823697	3.06697182
O	-0.46206964	8.13608180	3.06111571
C	0.44742725	5.47476529	3.95448210
H	0.44637563	4.55695748	4.54376791
H	-0.20851910	9.82455850	5.05335409
C	1.81113119	5.59825238	3.28535555
H	2.60838990	5.77664391	4.00498987
H	2.03634779	4.69186762	2.72069585
H	-0.29681977	5.32377748	3.16482184
O	1.83448935	6.64938205	2.31918699
O	2.45130843	7.79688509	2.88633250
H	-0.10601126	7.42131954	2.51437271
H	0.10216162	6.50058829	5.90471296

Sum of electronic and zero-point Energies=	-421.950083
Sum of electronic and thermal Energies=	-421.940966
Sum of electronic and thermal Enthalpies=	-421.940022
Sum of electronic and thermal Free Energies=	-421.983722

TS

(ENOL_13 to 2-Pentenal + H_2O_2)

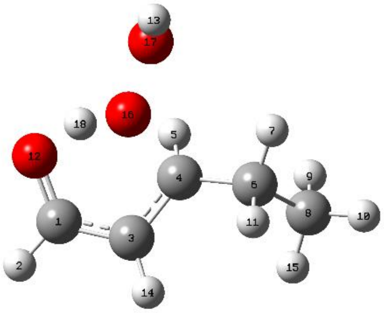

C	-1.68565476	-1.32749239	0.08775719
H	-2.31358576	-2.19139859	0.32169720
C	-0.33355052	-1.32462303	0.41061360
C	0.41672309	-0.23985778	-0.07348869
H	0.22332358	0.06819963	-1.09916679
C	1.79699430	0.04859261	0.40908865
H	2.01284509	1.10138237	0.22757972
C	2.80802727	-0.82449503	-0.34005580
H	2.74429393	-0.65629842	-1.41495909
H	3.82139602	-0.58987236	-0.01933879
H	1.85602753	-0.13390486	1.48184558
O	-2.25037242	-0.30957956	-0.41715681
H	-0.32202250	3.04734278	0.10416303
H	0.07535448	-2.02014419	1.12833465
H	2.62393831	-1.88124918	-0.15271904
O	-0.61361396	1.24283500	0.46862685
O	-0.35688164	2.27620804	-0.47276710
H	-1.47324704	0.68939498	0.01096064

Sum of electronic and zero-point Energies= -421.900700
Sum of electronic and thermal Energies= -421.891412
Sum of electronic and thermal Enthalpies= -421.890468
Sum of electronic and thermal Free Energies= -421.934920

2-Pentenal

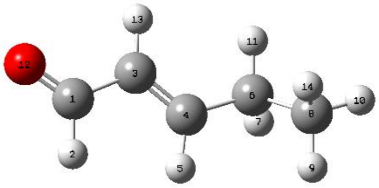

C	2.09218654	0.11923765	-0.15684848
H	2.17838134	-0.57907968	0.69852209
C	0.73393259	0.61448288	-0.41438471
C	-0.28410362	0.22765120	0.35027478
H	-0.07764221	-0.46790874	1.16259400
C	-1.70788859	0.64075835	0.18522870
H	-2.02809396	1.16325463	1.09053998
C	-2.62084519	-0.56546666	-0.04352447
H	-2.54824030	-1.26991650	0.78531075
H	-3.65992839	-0.25266432	-0.13034471
H	-1.79244222	1.34604022	-0.64152417
O	3.06165417	0.42449244	-0.80257626
H	0.60860875	1.30021378	-1.24443491
H	-2.34319988	-1.09096026	-0.95632658

Sum of electronic and zero-point Energies= -270.403740
Sum of electronic and thermal Energies= -270.396698
Sum of electronic and thermal Enthalpies= -270.395754
Sum of electronic and thermal Free Energies= -270.434813

H_2O_2 (see Figure 2)

TS$_{enol}$

(ENOL_24 to *cis*-Pentenone + H_2O_2)

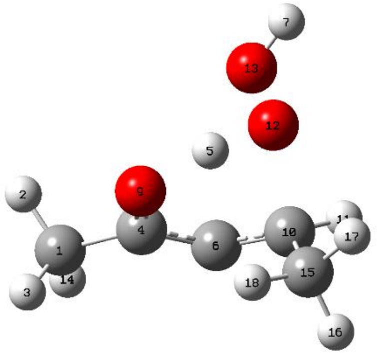

C	0.32012838	9.01448944	5.46895438
H	-0.58276086	9.57798838	5.23696201
H	1.16672773	9.69139816	5.34923941
C	0.47215529	7.89033314	4.49190146
H	0.02538781	7.09568736	2.65735593
C	0.53456877	6.55408751	4.89950797
H	-2.15032910	5.65292348	2.26697815
H	0.11564842	6.27578894	5.85461859
O	0.45070880	8.18322125	3.25233375
C	0.80954597	5.57501580	3.93558843
H	0.44649622	4.57341423	4.14577535
O	-0.34239169	6.04447015	2.45937196
O	-1.67171455	6.13732633	2.94856941
H	0.28777397	8.65883984	6.49523474
C	2.03413011	5.60716350	3.06897554
H	2.82983405	5.11081196	3.62802580
H	1.88210289	5.05825903	2.14161906
H	2.35034079	6.62379749	2.85315004

Sum of electronic and zero-point Energies=	-421.908241
Sum of electronic and thermal Energies=	-421.898730
Sum of electronic and thermal Enthalpies=	-421.897786
Sum of electronic and thermal Free Energies=	-421.942391

***cis*-Pentenone** (see Figure 2)

Ignition Delay Times (IDT)

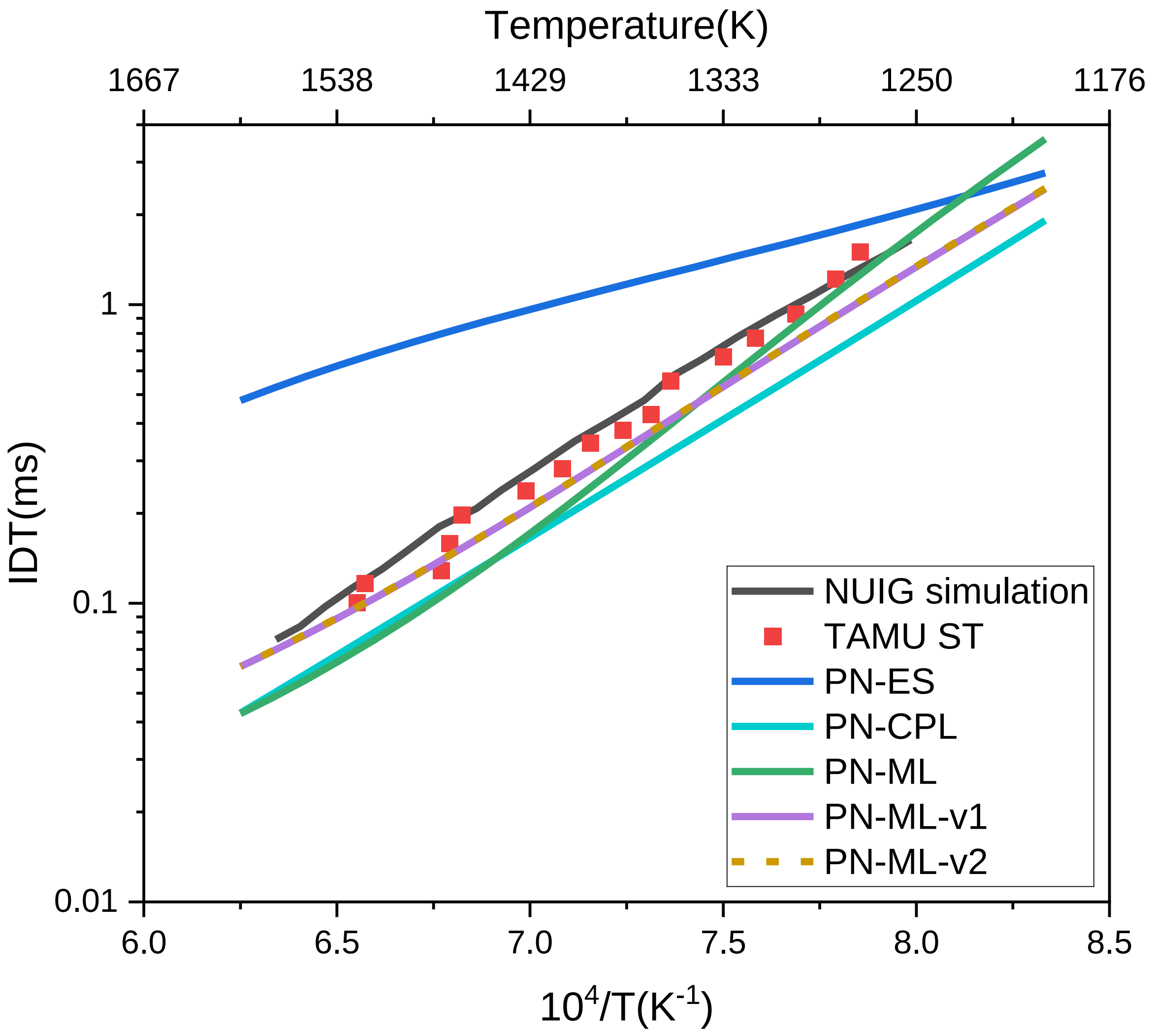

Figure S1. Effect of the use of new thermochemical library and kinetic families on model performance (ignition delay times - IDT) for $\phi = 2$ and $P = 1$ atm: using no libraries, only estimation methods (PN-ES), using only the Curran Pentane Library (PN-CPL), employing higher fidelity data via multiple libraries (PN-ML), employing higher fidelity data via multiple libraries and our modified reactions (PM-ML-v1), from our previous work [21], and employing higher fidelity data via multiple libraries and our modified reactions with addition of new thermochemical library and three kinetic families (PN-ML-m-v2). Symbols are experimental data; lines are model predictions.

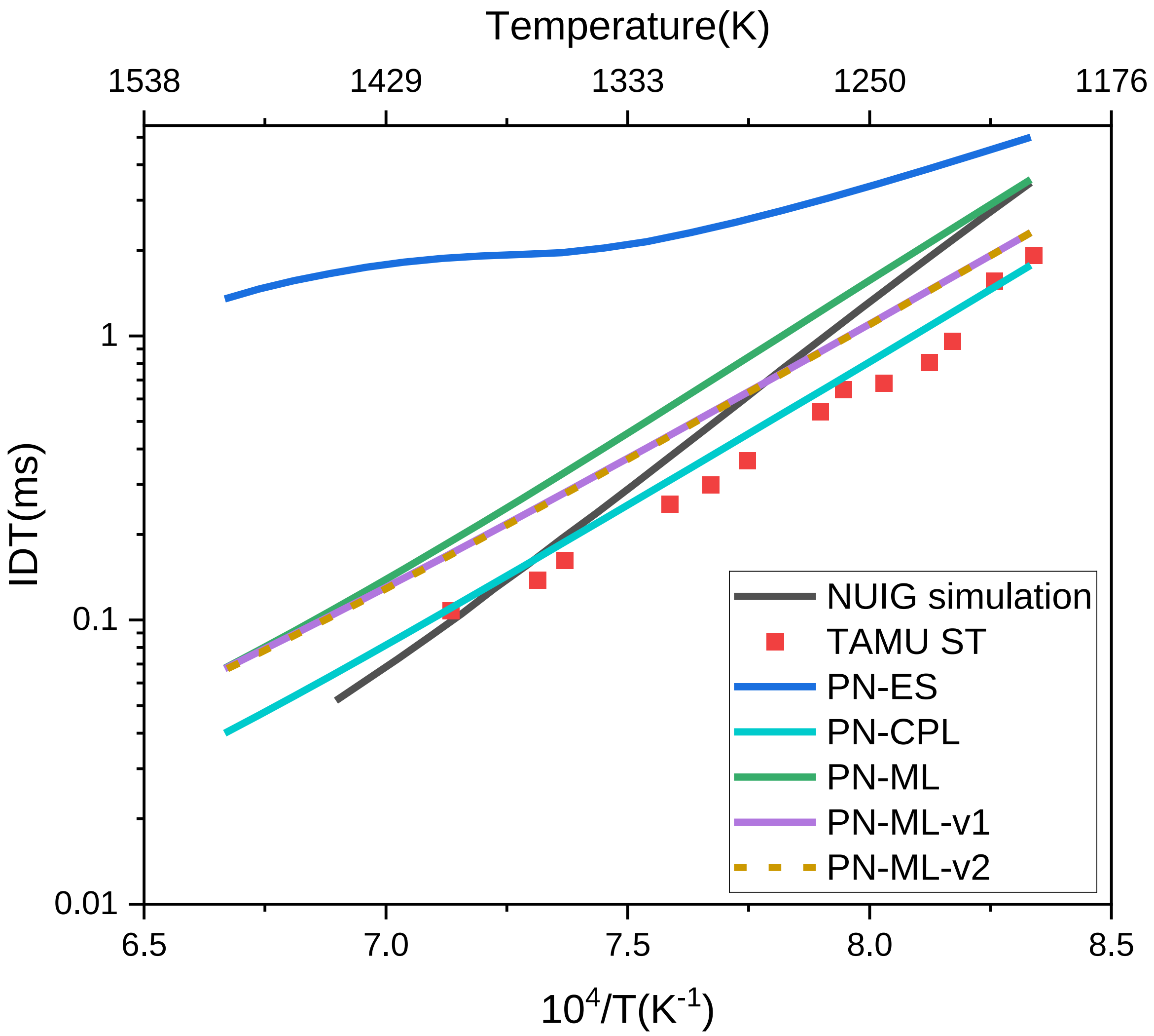

Figure S2. Effect of the use of new thermochemical library and kinetic families on model performance (ignition delay times - IDT) for $\phi = 0.5$ and $P = 1$ atm: using no libraries, only estimation methods (PN-ES), using only the Curran Pentane Library (PN-CPL), employing higher fidelity data via multiple libraries (PN-ML), employing higher fidelity data via multiple libraries and our modified reactions (PM-ML-v1), from our previous work [21], and employing higher fidelity data via multiple libraries and our modified reactions with addition of new thermochemical library and three kinetic families (PN-ML-m-v2). Symbols are experimental data; lines are model predictions.

Complete References

Reference 17:
Wang, Z.; Popolan-Vaida, D. M.; Chen, B.; Moshammer, K.; Mohamed, S. Y.; Wang, H.; Sioud, S.; Raji, M. A.; Kohse-Höinghaus, K.; Hansen, N.; Dagaut, P.; Leone, S. R.; Sarathy, S. M. Unraveling the structure and chemical mechanisms of highly oxygenated intermediates in oxidation of organic compounds. *Proc. Natl. Acad. Sci. U.S.A.* **2017**, *114*, 13102−13107.

Reference 98:
Dana A.G., Johnson M.S., Allen J.W., Sharma S., Raman S., Liu M., Gao C.W., Grambow C.A., Goldman M.J., Ranasinghe D.S., Gillis R.J., Payne A.M., Li Y.-P., Dong X., Spiekermann K.A., Wu H., Dames E.E., Buras Z.J., Vandewiele N.M., Yee N.W., Merchant S.S., Buesser B., Class C.A., Goldsmith C.F., West R.H., Green W.H. Automated reaction kinetics and network exploration (Arkane): A statistical mechanics, thermodynamics, transition state theory, and master equation software, *Int. J. Chem. Kin.* **2023**, *55*, 300-323.

Reference 118:
Gaussian 16, Revision A.03, M. J. Frisch, G. W. Trucks, H. B. Schlegel, G. E. Scuseria, M. A. Robb, J. R. Cheeseman, G. Scalmani, V. Barone, G. A. Petersson, H. Nakatsuji, X. Li, M. Caricato, A. V. Marenich, J. Bloino, B. G. Janesko, R. Gomperts, B. Mennucci, H. P. Hratchian, J. V. Ortiz, A. F. Izmaylov, J. L. Sonnenberg, D. Williams-Young, F. Ding, F. Lipparini, F. Egidi, J. Goings, B. Peng, A. Petrone, T. Henderson, D. Ranasinghe, V. G. Zakrzewski, J. Gao, N. Rega, G. Zheng, W. Liang, M. Hada, M. Ehara, K. Toyota, R. Fukuda, J. Hasegawa, M. Ishida, T. Nakajima, Y. Honda, O. Kitao, H. Nakai, T. Vreven, K. Throssell, J. A. Montgomery, Jr., J. E. Peralta, F. Ogliaro, M. J. Bearpark, J. J. Heyd, E. N. Brothers, K. N. Kudin, V. N. Staroverov, T. A. Keith, R. Kobayashi, J. Normand, K. Raghavachari, A. P. Rendell, J. C. Burant, S. S. Iyengar, J. Tomasi, M. Cossi, J. M. Millam, M. Klene, C. Adamo, R. Cammi, J. W. Ochterski, R. L. Martin, K. Morokuma, O. Farkas, J. B. Foresman, and D. J. Fox, Gaussian, Inc., Wallingford CT, **2016**.
